# Living in Heterogeneous Woodlands – Are Habitat Continuity or Quality Drivers of Genetic Variability in a Flightless Ground Beetle?

**DOI:** 10.1371/journal.pone.0144217

**Published:** 2015-12-07

**Authors:** Tamar Marcus, Steffen Boch, Walter Durka, Markus Fischer, Martin M. Gossner, Jörg Müller, Ingo Schöning, Wolfgang W. Weisser, Claudia Drees, Thorsten Assmann

**Affiliations:** 1 Institute of Ecology, Leuphana University Lüneburg, Lüneburg, Germany; 2 Institute of Plant Sciences and Botanical Garden, University of Bern, Bern, Switzerland; 3 Department of Community Ecology, UFZ Centre for Environmental Research, Halle, Germany; 4 Terrestrial Ecology Research Group, Department of Ecology and Ecosystem Management, Technische Universität München, Freising-Weihenstephan, Germany; 5 Institute of Biochemistry and Biology, University of Potsdam, Potsdam, Germany; 6 Max-Planck Institute for Biogeochemistry, Jena, Germany; 7 Zoological Institute, Biocenter Grindel, University of Hamburg, Hamburg, Germany; Embrapa, BRAZIL

## Abstract

Although genetic diversity is one of the key components of biodiversity, its drivers are still not fully understood. While it is known that genetic diversity is affected both by environmental parameters as well as habitat history, these factors are not often tested together. Therefore, we analyzed 14 microsatellite loci in *Abax parallelepipedus*, a flightless, forest dwelling ground beetle, from 88 plots in two study regions in Germany. We modeled the effects of historical and environmental variables on allelic richness, and found for one of the regions, the Schorfheide-Chorin, a significant effect of the depth of the litter layer, which is a main component of habitat quality, and of the sampling effort, which serves as an inverse proxy for local population size. For the other region, the Schwäbische Alb, none of the potential drivers showed a significant effect on allelic richness. We conclude that the genetic diversity in our study species is being driven by current local population sizes via environmental variables and not by historical processes in the studied regions. This is also supported by lack of genetic differentiation between local populations sampled from ancient and from recent woodlands. We suggest that the potential effects of former fragmentation and recolonization processes have been mitigated by the large and stable local populations of *Abax parallelepipedus* in combination with the proximity of the ancient and recent woodlands in the studied landscapes.

## Introduction

Today, one of the main goals of nature conservation is to maintain biodiversity. This is becoming ever more crucial given the current biodiversity crisis, which is being fuelled by the rapid changes in climate and in land use. Genetic diversity is an essential component of biodiversity, and while much work has been done on species diversity [1–3], fewer studies focus on the genetic level and its drivers. Genetic diversity is considered crucial for species' survival [4–6] as it serves as the basis for short-term and long-term processes of evolution and adaptation, allowing species to cope with changes such as those in climate and land use [7,8]. In addition, genetic diversity has been shown to enhance population fitness [9,10], to enhance resistance to parasites [11], and to stabilize population dynamics [4,5].

Previous studies have uncovered a complex network of factors that drive genetic structure, in the sense of genetic diversity together with genetic differentiation, including landscape parameters [12,13], population history and size [13,14], habitat history [15,16], and environmental drivers such as temperature [14]. These factors affect genetic structure either directly via selection, or more often indirectly via population sizes and gene flow, and can cause changes both in genetic diversity within groups of individuals, and in genetic differentiation between such groups. Variations in the landscape, in population size and structure, and in the environment can be thought of in essence as changes in habitat stability and in habitat suitability across time and space. More suitable habitats usually have larger populations which are less affected by genetic drift. Populations in more stable habitats have experienced less founder effects and bottlenecks, and have also had more time to accumulate alleles due to migration and possibly to mutation. In addition, large scale genetic patterns may exist as a result of long-term processes such as post-glacial recolonization [17].

A common study focus is the effect of habitat continuity and other historical factors on genetic structure, especially in the context of ancient and recent woodlands in Central and Western Europe or in North America (e.g. [13,16,18,19]. In these regions, ancient woodlands are defined as areas that have been wooded continuously since the earliest accurate, comprehensive maps of the area are available, in the case of Europe usually around 200–400 years ago. This is approximately the same time frame when peak fragmentation of the forests is thought to have occurred [20]. Although Central Europe is naturally covered by woodlands, most of the contemporary ones are consequences of afforestation or of natural succession that occurred on cleared or managed sites that were then subsequently abandoned. These changes can often be identified using chronological sequences of historical maps, and such woodlands are known as recent woodlands [21,22].

While studying ancient and recent woodlands sheds light on long-term historical processes, an additional way to look at habitat continuity in forests is to examine stand age, which reflects a short-term definition of site history. Stand age refers to the age of the current trees, regardless of which habitat was there previously. Studies of the effects of habitat continuity on genetic diversity tend to find higher diversity in the ancient woodlands than recent ones [16,23–25], and older stands tend to be more genetically diverse than younger stands [15,26]. In either case, the higher genetic diversity in the longer-term habitats is explained by the habitat stability and the greater resulting suitability for many woodland species.

Woodlands that were clear cut and then replanted immediately may still be considered ancient woodlands, although clear cutting can strongly alter biotic and abiotic properties of a woodland such as light availability, microclimate, and habitat structure (e.g. [27,28]). The litter and soil layers, which serve as the main habitat for many ground beetles and their prey, are also sharply affected by clear cuts. These changes can include amongst others, changes in layer thicknesses, chemical composition, and structure (e.g. [29–32]).

Although both environmental (e.g. [14,33,34]) and historical variables have been shown to affect genetic diversity (e.g. [16,18,35]), few studies examine their effects jointly. This is especially surprising as many environmental variables, especially those connected to soil, may have complex relationships with genetic diversity as they reflect the history of a site as well as affecting current population sizes [30,36]. Additionally, most studies have concentrated on rare species or on very fragmented habitats where, unsurprisingly due to the higher probability of stochastic effects, strong genetic effects have been found (e.g. [18,19,23,24]). Much less is known about the drivers of genetic diversity in more common species in woodland habitats that contain patches of varying ages and sizes, although this is a widespread landscape structure both in Europe and in parts of North America. Therefore, it is not known if habitat history is expected to shape the genetic structure of typical woodland species in these landscapes.

*Abax parallelepipedus* (Piller & Mitterpacher, 1783), a flightless, forest-dwelling ground beetle [37], is a widespread species in Central European woodlands and is an interesting test case in the context of the study of genetic diversity drivers (S2 Fig). On the one hand it is known that fragmentation can cause extremely rapid, significant changes in the genetic structure of this species [38] and it is both flightless as well as restricted to wooded habitats. On the other hand it can reach high, stable population densities of approximately 0.23 individuals/m^2^ [39] during the main activity period even in small habitat patches [38,40], which is expected to stabilize genetic structure.

We examined the possible effects of both historical and current parameters on genetic diversity in *Abax parallelepipedus*. The study was carried out in two regions in Germany, in the Schorfheide-Chorin and in the Schwäbische Alb, which both have a mosaic of varied land uses and are fragmented but not overly so. Our study sites, part of the Biodiversity Exploratories research platform, represent a wide range of the environmental conditions and land uses present in each region [41]. We analyzed a set of 14 microsatellite loci in 24 individuals each from 88 plots that are located in a mosaic of ancient and recent woodlands with varying stand ages in both regions. We addressed the following main questions: (i) What are the drivers of genetic diversity in *Abax parallelepipedus*? (ii) Is there significant genetic differentiation between local populations found in ancient and recent woodlands? (iii) Does land use intensity affect genetic diversity due to expected changes in local population sizes?

## Methods

### Study area and plot selection

**Fig 1:**
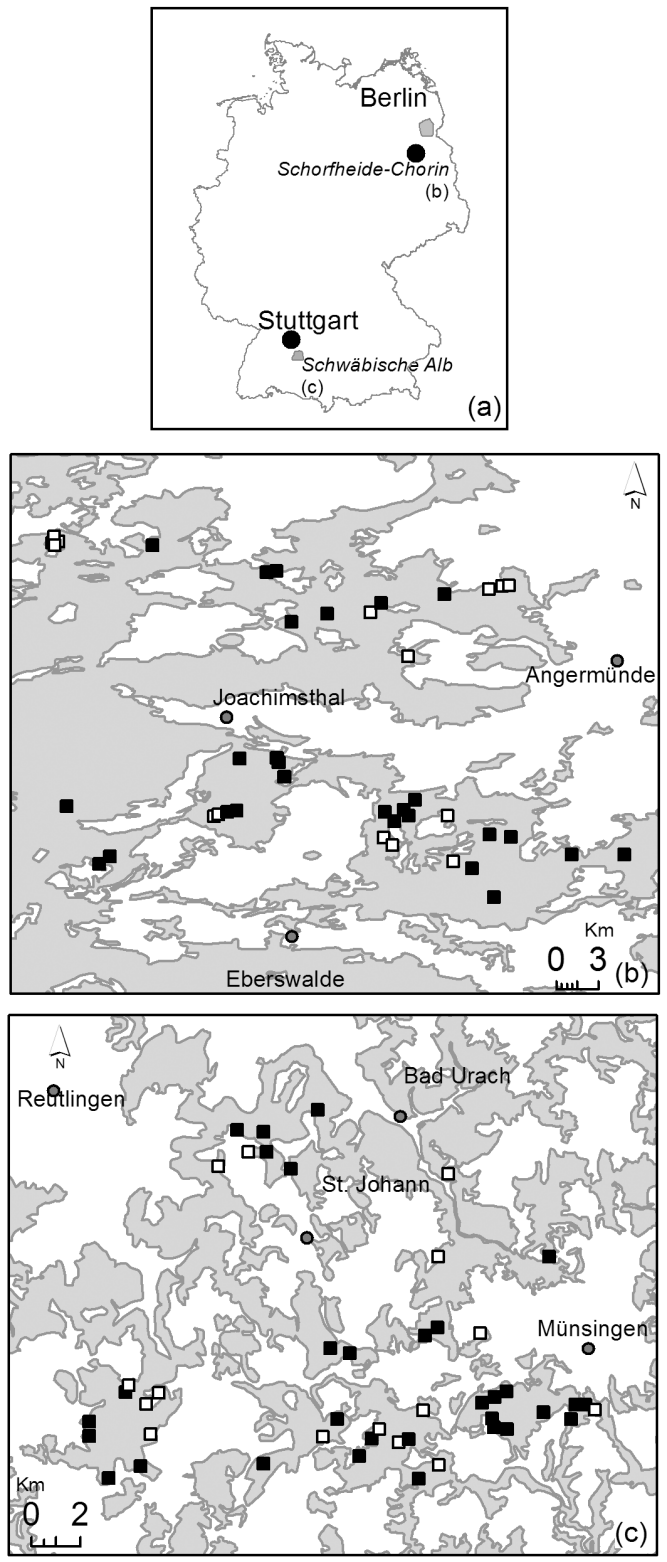
Map of research sites. (a) Location of study regions in Germany. Distribution of plots in woodlands (grey areas) in the Schorfheide-Chorin (b) and in the Schwäbische Alb (c). Woodlands defined as per the Corine Land Cover 2006 dataset [42]. Open boxes are plots located in recent woodlands, closed boxes are located in ancient woodlands. Small dots indicate named towns and villages. Note that the scales of the maps are different. All maps were created using ArcGIS ver. 10.1 [43].

In the springs and summers of 2011–2012 we sampled *Abax parallelepipedus* from the Schwäbische Alb (southwestern Germany; n = 46) and the Schorfheide-Chorin (northeastern Germany; n = 42) in the 100 m x 100 m forest plots of the "Biodiversity Exploratories" (Fig 1A). The forest plots in each region represent the forest types commonly found in the regions, and include both unmanaged forests and age class forests. Age class forests result from clear cuts, usually small scale ones, or from shelterwood logging in which trees are removed in two rounds. In the first cut of shelterwood logging, most of the stand is cleared leaving some trees standing to shelter seedlings. These remaining trees are then cut in a second round after the young trees have created a canopy layer. Stands of European beech (*Fagus sylvatica*), pedunculated and sessile oak (*Quercus robur* and *Q*. *petraea*), and Scots pine (*Pinus sylvestris*) are found in the Schorfheide-Chorin, while the Schwäbische Alb is dominated by stands of European beech and Norway spruce (*Picea abies*). Some of the stands are monodominant while others are mixed stands.

The plots were selected in a two stage process ensuring that the plots represent the gradient of forestry management practices, their intensities, and soil characteristics for the most common soil types in the given region (see S2 Table and [41]). First, 500 potential plots that reflect the common forest types were selected for each region. Then 50 plots were selected in each region from the pools using stratified random sampling. The plots are randomly distributed within the regions (Fig 1B and 1C, for plot numbers see S1 Fig), are located at least 200 m from another plot and at least 100 m from the nearest forest edge. The large number of plots per region allow for a thorough representation of environmental parameters found in each region. For more details on plot selection, see Fischer et al. [41].

### Study species

The flightless ground beetle *Abax parallelepipedus* is strictly limited to forests, and inhabits the litter layer [37,44,45]. The species is known to have large, stable populations [39,46,47] and is known to prefer ancient woodlands in some regions, such as the lowlands of northwestern Germany and Belgium [48,49]. The species is considered a forest generalist and can be found in large numbers in both conifer and broadleaf forests of varying ages, including conifer plantations [50–54]. Its dispersal power was found to be low, moving on average between 0.6 m and 2.3 m per night (reviewed in [55]).

### Sample collection and microsatellite genotyping

We collected beetles by using ten live pitfall traps per plot baited with red wine on cellulose during the spring and summer of 2011 (Schwäbische Alb) and of 2012 (Schorfheide-Chorin). In all plots, the traps were placed in a straight line, 10 m apart along the plot border to ensure equal sampling area. We gathered all of the *Abax parallelepipedus* individuals we found in the traps approximately once a week and rebaited the traps until we had caught 33 individuals in the plot. We pooled the beetles trapped in all the traps of a plot each collection round and froze them at-80°C. Field work permits were issued by the responsible state environmental offices of Baden-Württemberg and of Brandenburg (according to §72 BbgNatSchG).

We extracted DNA using the CTAB extraction protocol [56] from three legs from each of 24 randomly selected beetles for each plot. We genotyped 14 polymorphic microsatellite loci using an ABI 3730 Genetic Analyzer (Applied Biosystems, Foster City, CA, USA). For PCR, sequencing protocols, and information about the loci see [57].

Deviation from Hardy-Weinberg Equilibrium (HWE) was tested using GENEPOP 4.2 [58] and no significant deviation was found (percentage of local populations not in HWE: Schwäbische Alb = 5.288%, Schorfheide-Chorin = 4.240%). Suspected presence of null alleles was tested using MICRO-CHECKER 2.2.3 [59] and no null alleles were found. Linkage disequilibrium (LD) was checked using FSTAT 2.9.3.2 [60,61]. No significant LD was found. Allelic richness, the rarefied numbers of alleles as a measure of genetic diversity, was calculated for each plot using FSTAT 2.9.3.2. The rarefaction was done per local population, with a minimum sample size of 20 individuals, to account for isolated instances of ineffective PCR reactions.

Overall F_ST_ values among local populations, a measure of genetic differentiation, were calculated for each region using Arlequin 3.5.1.3 [62]. Private alleles, meaning those found in only one local population, and unique alleles, meaning those found in a specific group of plots either by region, by ancient or recent woodlands, or by population density, were counted and tallied. The grouping by local population density was done by grouping plots into percentiles based on the number of individuals caught in the 2008 killing traps (see S1 Text). Genetic clustering was tested using the algorithm developed by Pritchard [63] as implemented in STRUCTURE 2.3.4 for each region separately, to ensure that no underlying clustering is affecting the results. In this analysis we used the admixture model with no use of previous information about sampling location. Burnin length was 20,000 and there were 100,000 MCMC repeats after burnin. Number of clusters was run from K = 1 to K = the number of plots+1 for the Schorfheide-Chorin (K = 43), and for the Schwäbische Alb (K = 47). For the Schwäbische Alb for higher values of K, the runtime was insufficient to find proper solutions, and therefore a second run of K = 1 to K = 30 was analyzed (S3B Fig). We used CLUMPAK [64] and HARVESTER [65] to find the most likely K using the Evanno method [66] and to visualize the results.

### Plot characterization

We characterized each plot in terms of variables related to the litter layer, which serves as the beetle's habitat, in terms of variables that can be related to the land use history of the plot, in terms of variables that can be related to local population size, and also in terms of variables related to soil, vegetation, climate, geography, and forest management. To characterize our plots in terms of general parameters known to affect ground beetles and therefore *Abax parallelepipedus* (for general overview see [67], with specific references listed for each variable), we used longitude [68], latitude [69], elevation [70,71], mean annual temperature [71,72], mean annual precipitation [71], forest management type [53], main tree species [53], number of vascular plant species [51,73], soil type [74], soil pH [75], and the Forest Management Intensity index (FORMI) ([53], defined in [76]). Depth of the litter layer and ground cover of litter, of deadwood, and of trees (see [77,78]) were included to characterize the habitat of the beetle and thus local population sizes. Land use history was characterized by defining each plot as an ancient or a recent woodland (see below), by stand age, and by the percentage of closed forest species. The depth of the O_e_ soil layer, as well as the C/N ratio of the O_i,_ the O_e_, and the A soil layers and the carbon content of the A layer were also included as they are known to reflect historical land use [32,36]. As local population sizes could not be determined directly, we used three proxies as estimates. The first proxy is the percentage of forested landscape in the two kilometers surrounding each plot, the second is the sampling effort needed to collect 33 individuals in 2011 or 2012, and third is the number of *Abax parallelepipedus* trapped in killing traps in 2008 (details found in S1 Text).

Note that sampling effort is expected to be negatively correlated to local population density as the less dense a local population is, the longer it should take to collect 33 individuals. More details on all the variables and on their collecting methods can be found in S2 Table and in S1 Text.

Ancient woodlands are defined as areas that appear as covered by trees over the complete time series of existing sufficiently accurate maps [20,79]. For the Schwäbische Alb, plots were defined as either *ancient* or *recent* based on eight maps dating from 1820 and onwards (S1 Table) (n_ancient_ = 31, n_recent_ = 15). For the Schorfheide-Chorin, plots were defined as either *ancient* or *recent* based on four maps dating from 1767 and onwards (S1 Table) (n_ancient_ = 26, n_recent_ = 16). Any plot that appears as non-wooded on at least one map was defined as recent woodland. All others were defined as ancient woodlands. Stand ages were taken from the latest forestry inventory available [80]. In plots with trees of more than one age class, stand ages were defined as the age of the older age class (S2 Table).

### Statistical analyses

We modeled the relationship between plot characteristics, including measures of habitat continuity as well as environmental parameters, and allelic richness. We started with 27 predictor variables (S2 Table). We first tested for collinearity between the predictor variables and removed the smallest number of variables possible while eliminating all instances where Spearman's rho > |0.7| [81], leaving us with 18 predictors (S3 Table). When we could not choose which variable to eliminate based on maximizing the number of remaining variables, we chose to retain the one more correlated with allelic richness. We created a general linear model for each region using allelic richness as a response variable. We modeled the regions separately as the means and variances of allelic richness are different due to the environmental conditions and history of the regions. The models were reduced using a backwards step reduction process based on AICc scores. The models with the lowest AICc scores, and the smallest numbers of predictors in the case of ΔAICc<2 between two models, were selected (see [82]). The residuals were checked to ensure that they are normally distributed and the residuals were plotted against the fitted values to investigate homogeneity of variance. As stand age and the FORMI index are significantly correlated (Spearman Rank Correlation: rho = -0.700, p<0.001, S3 Table) and we were interested in testing both of these parameters, we ran these models twice, once using stand age and once using FORMI as a possible explanatory variable.

We tested for spatial autocorrelation using Moran's I both of the allelic richness values themselves for each region using the APE package [83] and of the residuals of the model using the ncf package [84] and corrected using Bonferroni's correction for multiple testing. We examined the effects of long-term habitat continuity on genetic differentiation using two methods. We first ran an AMOVA in Arlequin 3.5.1.3 [62] for each region separately, grouping the plots by whether they are located in an ancient or in a recent woodland. We then tested the effects of stand age, of location in ancient or in recent woodlands, and of the interaction between them on genetic differentiation using GESTE 2.0 [85]. This program uses hierarchical Bayesian methods to find population-specific F_ST_ values, which are then modeled with the historical variables we provided in a generalized linear model. GESTE was run using default parameters. If not otherwise stated, all statistical analyses were done using R 3.0.0 [86].

## Results

We analyzed 2112 individuals from 88 local populations and found 71 alleles across the 14 loci. Numbers of alleles (A) per locus ranged from 3–13 alleles with a mean of 5.1 (Table 1). All loci were polymorphic. For local level population genetics statistics see S4 Table. Mean allelic richness across all local populations was 1.96 alleles per locus. The allelic richness was not spatially autocorrelated in either region (Moran's I: Schwäbische Alb p = 0.439, Schorfheide-Chorin p = 0.535). The sampling effort in the Schorfheide-Chorin in number of days needed to collect 33 individuals (range = 8–80 days, mean = 46±20 days) was higher than that in the Schwäbische Alb (range = 5–27 days, mean = 16±7 days) (Wilcoxon rank sum test: W = 200.5, p<0.001). No evidence of spatial genetic clustering was found for either region as two and three gene pools were identified for the Schorfheide-Chorin and for the Schwäbische Alb respectively, which however, were largely admixed within individuals and mixed across most of the populations (Schwäbische Alb: S3 Fig; Schorfheide-Chorin: S4 Fig; for distribution of individuals belonging to each cluster see S1 Fig; plot numbers in S3 and S4 Figs refer to map found in S1 Fig).

**Table 1:** Distribution of alleles in recent and ancient woodlands of two regions in Germany. In total 72 alleles were found.

Region	Group	Total number of alleles* (range per plot)	Unique alleles**	Private alleles***
Schwäbische Alb	All	68 (30–38)	24	9
	Ancient woodlands (n = 31)	62 (30–37)	9	5
	Recent woodlands (n = 15)	56 (30–38)	5	4
Schorfheide-Chorin	All	47 (18–30)	4	3
	Ancient woodlands (n = 27)	40 (18–30)	1	1
	Recent woodlands (n = 15)	40 (19–29)	3	2

*not rarefied

**found in one region only

***found only in a single plot.

There were 24 (34%) alleles that occurred only in the Schwäbische Alb, and four (6%) only in the Schorfheide-Chorin (Table 1). When the local populations from ancient woodlands and the recent woodlands were pooled regardless of region, 12 alleles (17%) were found only in ancient woodlands, and eight alleles (11%) occurred exclusively in recent woodlands. We found 6 (8%) private alleles, meaning alleles found only in one local population, both for ancient woodlands and for recent woodlands. For all allele frequencies, see S4 Table. When plots were grouped based on the number of *Abax parallelepipedus* individuals found in the 2008 killing traps, the highest number of private alleles was found in the plots with the lowest local population density (for details by region see Table 2).

**Table 2:** Number of private alleles in classes of local population density.

Region	0-20^th^ percentile	21^st^-40^th^ percentile	41^st^-60^th^ percentile	61^st^-80^th^ percentile	81^st^-100^th^ percentile
Schwäbische Alb	7	2	1	0	2
Schorfheide-Chorin	2	1	3	2	1
Both regions	5	1	3	1	2

Categories are based on number of *Abax parallelepipedus* individuals found previously using killing traps in 2008 (see S1 Text).

In order to understand the drivers of genetic diversity, we modeled the effects of habitat continuity and stand age together with an additional 16 habitat, soil, vegetation, and local population variables on allelic richness (S2 Table). For the Schwäbische Alb, the only variable to remain in the model was the percentage of the two kilometers surrounding each plot that is forested which had a negative effect (Table 3), though the effect on allelic richness was borderline significant. In the Schorfheide-Chorin region, genetic diversity was positively affected by the depth of the litter layer (p = 0.018) and by the sampling effort (p = 0.009) (Table 3). We found no effects of land use history in any of our models, neither of stand age nor of habitat continuity. As stand age fell out of the models in the initial reduction step, replacing stand age with land use intensity (FORMI index) gave the same results as shown in Table 3. The residuals of the models showed no spatial autocorrelation (S5 Fig).

**Table 3:** Variables that remained in the general linear model for each region.

Region	Variable or model information	Estimate±SE	t-statistic	p-value
Schwäbische Alb	Percentage of surrounding landscape (2 km radius) that is forested	-0.377±0.195	-1.938	0.059
	Initial/final AICc: -9.899/-59.299			
	Adjusted R^2^ = 0.056			
	F_(1,44)_ = 3.756, p = 0.059			
Schorfheide-Chorin	Depth of O_i_ layer	0.087±0.035	2.464	0.018
	Sampling effort	0.004±0.002	2.732	0.009
	Initial/final AICc: 59.258/-11.251			
	Adjusted R^2^ = 0.253			
	F_(2,39)_ = 7.949, p = 0.001			

Shown for each *variable* are the model estimates ± SE, t-values, and the p-value of the t-statistic. For each *model* initial and final AICc scores and adjusted R^2^ values are presented. See S6 and S7 Figs for genetic diversity plotted against each of the remaining variables as well as against the proxies of local population size for each of the two regions.

The level of overall differentiation in the study species was low, but was lower by an order of magnitude in the Schwäbische Alb (Schwäbische Alb: F_ST_ = 0.005, p = 0.002; Schorfheide-Chorin: F_ST_ = 0.047, p<0.001). The AMOVA grouping local populations collected from ancient or from recent woodland showed no significant differentiation for either region (Table 4), although local populations within groups were significantly differentiated. Modelling the relationship between historical variables and population-specific F_ST_ values also did not find any significant effects, as for both regions the model with the highest posterior probability was that which contained only a constant.

**Table 4:** Results of AMOVA comparing the genetic differentiation between local populations found in ancient and recent woodlands for each region.

	Schwäbische Alb				Schorfheide-Chorin			
	Sum of Squares	Variance component (p-value)	Percentage variation	Range of degrees of freedom	Sum of Squares	Variance component (p-value)	Percentage variation	Range of degrees of freedom
Among groups	1.322	-0.001 (p = 0.874)	-0.062	1	2.502	<0.001 (p = 0.336)	0.009	1
Among local populations within groups	109.428	0.011 (p<0.001)	0.547	44	97.441	0.036 (p<0.001)	4.673	40
Within local populations	4167.022	1.979 (p = 0.001)	99.515	2098–2110	1420.627	0.741 (p<0.001)	95.318	1902–1920
Total	4277.826	1.989			1520.570	0.778		

## Discussion

We found no relationship between either long-term or short-term habitat continuity and genetic diversity in the 88 local populations of *Abax parallelepipedus* from the Schwäbische Alb and from the Schorfheide-Chorin. For the Schwäbische Alb we did not find a significant effect of any of the 18 variables we tested on genetic diversity. For the Schorfheide-Chorin however, we found a significant effect of both the depth of the litter layer and required sampling effort, which serves as a proxy for local population density. We furthermore found no significant differentiation between local populations from ancient and recent woodlands in either region.

### Landscape history

Based both on the lack of differentiation between the local populations from ancient woodlands sites as well as on the similar levels of genetic diversity of those from the ancient and the recent woodlands, we can conclude that genetic structure (in the sense of a combination of genetic diversity and genetic differentiation) of *Abax parallelepipedus* sampled in the studied regions does not reflect historical processes. This strongly suggests the maintenance of relatively stable, large populations during the peak of fragmentation, as well as lack of founder effects and bottlenecks during recolonization processes. It is also possible that drift effects which may have occurred, have been mitigated by high levels of subsequent gene flow and recolonization from several source sites [87].

We found a similar lack of effect of stand age, which implies effective recolonization of woodlands after clear cuts, although clear cut areas prior to tree regrowth should not be a suitable habitat for our study species. *Abax parallelepipedus* is relatively immobile for a ground beetle of its size [88], nevertheless it still can move efficiently into newly planted woodlands from adjacent ones, especially as it is not restricted to a specific type of forest [40,50,89,90]. This mobility and forest generalist habitat requirements also explain the lack of effect of land use intensity. As forest management in Germany today generally consists of very small scale clear-cuts or of shelterwood forestry, the remaining, neighboring forests or the protective layer of trees can apparently sustain large enough populations of *A*. *parallelepipedus* to maintain genetic diversity.

Our results highlight, that although it is commonly accepted that ancient woodlands [20] and less fragmented landscapes are of greater conservation value due to their greater genetic diversity, this may not be true in all cases. Woodlands must be evaluated in the context of the overall structure of the surrounding landscape, and not on the basis of their site-based characteristics alone. In our study sites, the proximity of the recent woodlands to the ancient ones, combined with the relatively large fragment size of the ancient woodlands seems to have completely mitigated the effects of long-term and short-term changes in land use. Vandepitte et al. [13] found similar results in a study of the genetic structure of the herb *Geum urbanum* L., a species that also disperses fairly rapidly into new woodlands [91]. This study also took place in an area that contains ancient and recent woodlands in close proximity, so here too gene flow may be mitigating any historical effects at the genetic level.

Most other studies that found an effect of habitat continuity on genetic diversity, including the studies of *Carabus problematicus* Herbst and of *C*. *auronitens* Fabricius, sampled extremely fragmented woodlands (e.g. [23,24,25,92]). In these cases, the genetic traces of the fragmentation and recolonization processes would likely be stronger, as fewer individuals can migrate to the newly wooded patches leading greater, unmitigated founder effects and genetic erosion that persist due to low gene flow.

### Population size

**Fig 2:**
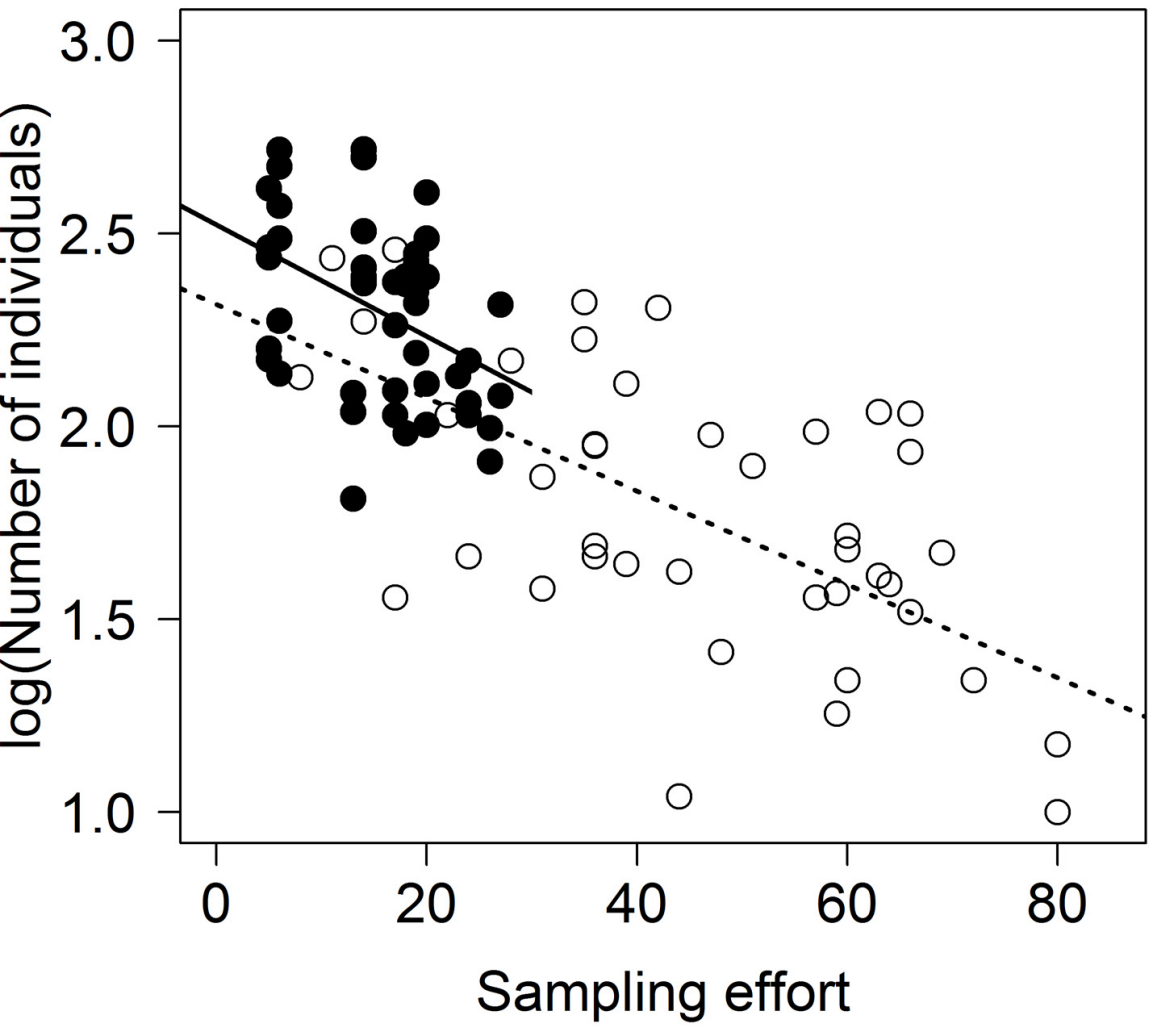
Comparison of sampling effort and number of individuals caught in previous pitfall trapping. Comparison of number of days required to catch 33 individuals of *Abax parallelepipedus* in our live traps and number of individuals caught in two pitfall traps per plot from April to October 2008 (S1 Text for details). Linear model shows a significant negative relationship in both regions (closed circles, solid line—Schwäbische Alb: estimate = -0.014±0.005, p = 0.003; open circles, dashed line—Schorfheide-Chorin: estimate = -0.012±0.003, p<0.001).

Another factor contributing to the stability of the genetic diversity and lack of genetic differentiation is the stability of the population sizes of *Abax parallelepipedus* [39,46]. As a result, the likelihood of populations undergoing changes in their genetic structure due to random effects caused by sudden drops in population size is lower (e.g. [93]). In a linear model we found a significant negative relationship between sampling effort and the log-transformed number of individuals collected in pitfall traps during 2008 (Fig 2; Schwäbische Alb: closed circles, solid line; Schorfheide-Chorin: open circles, dashed line; methods found in S1 Text). This not only justifies our use of sampling effort as a proxy for local population size, but also shows the stability of the local population sizes between the years as the pitfall trapping and our collecting were not carried out in the same years.

In the Schorfheide-Chorin we found significant, positive effects of the depth of the litter layer and of the sampling effort on genetic diversity. We interpret these results as representing effects of local population density on genetic diversity as sampling effort directly reflects local population density, while the depth of the litter layer reflects prey availability. Deeper litter layers contain more earthworms (Lumbricidae) [94–96] and thus can support larger local populations of *Abax parallelepipedus*, as they serve as a main food source for the beetles [67,97]. This highlights the importance of population size in preventing loss of alleles and preventing differentiation due to drift. We found a non-significant negative correlation between allelic richness and percentage of forest cover of the two kilometers surrounding each plot for *Abax parallelepipedus* in the Schwäbische Alb. The non-significance of this driver in the Schwäbische Alb is probably due to the extremely low variation in allelic richness found there.

At first, the direction of one of our results, namely the positive effect of sampling effort in the Schorfheide-Chorin, seems to contradict general population genetics theory in that lower local population densities seem to lead to higher genetic diversity. This may be due to the longer period of time required to gather 33 individuals in plots with lower local population densities. The longer trapping times potentially enabled individuals from more distant interaction groups (sensu [98,99]), thus with additional alleles to be sampled. An interaction group is a micro-scale, sub-plot level population structure that describes a group of individuals that live in an area small enough to ensure that they are likely to meet for reproductive purposes. As most individuals will reproduce with a member of their interaction group, members of an interaction group tend to be more similar genetically to each other than to individuals from farther away. The longer our traps were open, the better our chances to catch individuals from more distant interaction groups that likely have a slightly different genetic makeup resulting in an overall higher genetic diversity value. This hypothesis is corroborated by the fact that the plots with the lowest local population densities have the highest number of private alleles.

The generally low level of allelic richness, as well as the higher variability in the Schorfheide-Chorin may be explained by the lower local population sizes and densities relative to the Schwäbische Alb. We attribute this to the higher soil acidity of the Schorfheide-Chorin, which is known to lead to smaller earthworm populations [100], which serve as a main food source [67,97] for the beetle. This relationship between soil pH, prey, and population sizes of *Abax parallelepipedus* has been reported previously by Jukes et al. [69] and Magura et al. [75]. The sampling effort needed to trap 33 individuals in the Schorfheide-Chorin was higher than in the Schwäbische Alb, there were less individuals of *A*. *parallelepipedus*, and there was a significant correlation between sampling effort and pH in the Schorfheide-Chorin (linear model: estimate = -0.004±0.002, p = 0.045). These local populations with lower density are more susceptible to genetic drift and other stochastic processes, leading to the loss of alleles.

## Conclusions

We found a weak genetic structure in *Abax parallelepipedus*, a common forest species in a moderately fragmented landscape that is mainly driven by current rather than historical parameters. Under such conditions, sufficiently large population sizes and gene flow have so far either prevented or mitigated genetic effects of historical and current fragmentation. Although we found no effect of long-term or short-term habitat continuity on genetic diversity or differentiation, we do not question the conservation value of ancient woodlands and of old stands. While these properties may not be important drivers of genetic diversity in our study species and regions, history is an important driver in more fragmented regions such as Flanders [24,92] and in rarer species such as the lichen *Lobaria pulmonaria* [25,101]. Ancient woodlands and old stands also have importance for conserving species diversity as well as species that are restricted to these habitats [48,102]. In addition, our results reflect the importance of micro-scale population structures such as interaction groups, and highlight the need to account for such structures while examining historical as well as current drivers of genetic population structure. Finally, we emphasize the importance of a landscape approach to conservation, and the importance of ensuring proximity of ancient and more recent woodlands in order to allow both species and individuals with different alleles to effectively colonize new sites.

## Supporting Information

S1 Fig Map of plot numbers and of clustered individuals. (a) Schorfheide-Chorin, (b) Schwäbische Alb. Individuals in the Schorfheide-Chorin were assigned to the cluster to which they had larger than a 50% chance of belonging as per STRUCTURE. The pie charts present the number of individuals sampled from each local population belonging to each of the clusters. Replacing the number of individuals belonging to each cluster with the likelihood of belonging to each cluster gives a similar pattern. Grey areas are forested (see legend of Fig 1). Squares indicate named towns and villages. Note that the scales of the maps are different. All maps were created using ArcGIS ver. 10.1 [43].
(TIF)

S2 Fig Photograph of *Abax parallelepipedus*. (TIF)

S3 Fig Results of STRUCTURE analysis–Schwäbische Alb. (a) ΔK/K plot, (b) mean likelihood and variance for each K–for K = 1 to K = 47, (c) mean likelihood and variance for each K–for K = 1 to K = 30, (d) membership probability of individuals for K = 3.
(TIF)

S4 Fig Results of STRUCTURE analysis–Schorfheide-Chorin. (a) ΔK/K plot, (b) mean likelihood and variance for each K–for K = 1 to K = 43, (c) membership probability of individuals for K = 2.
(TIF)

S5 Fig Correlograms of model residuals. Empty circles indicate non-significant values.
(TIF)

S6 Fig Rarefied allelic richness and important variables–Schwäbische Alb. Relationship between rarefied allelic richness and proxies of population size and depth of the litter region for the Schwäbische Alb. Results are similar to those of the reported models (Spearman Rank Correlation; forested surrounding landscape: rho = -0.169, p = 0.261, sampling effort: rho = 0.088, p = 0.562, individuals from killing traps: rho = -0.286, p = 0.054, depth of litter layer: rho = 0.012, p = 0.935).
(TIF)

S7 Fig Rarefied allelic richness and important variables–Schorfheide-Chorin. Relationship between rarefied allelic richness and proxies of population size and depth of the litter region for the Schorfheide-Chorin. Results are similar to those of the reported models (Spearman Rank Correlation; forested surrounding landscape: rho = 0.048, p = 0.761, sampling effort: rho = 0.397, p = 0.009, individuals from killing traps: rho = -0.212, p = 0.177, depth of litter layer: rho = 0.421, p = 0.005).
(TIF)

S1 Table Maps used to define woodlands as recent or ancient. (PDF)

S2 Table Full list of variables used to characterize plots. (PDF)

S3 Table Collinearity matrix of continuous predictor variables for variable selection (Spearman's rho values). Values greater than |0.7| are marked in bold.
(PDF)

S4 Table Allele frequencies and related indices. (PDF)

S1 Text Supplementary methods. (DOCX)
