# Supplementary figures and images for: Living in Heterogeneous Woodlands – Are Habitat Continuity or Quality Drivers of Genetic Variability in a Flightless Ground Beetle?
Source: PLoS One. 2015 Dec 7;10(12):e0144217. doi: 10.1371/journal.pone.0144217 (PMC4671619; doi:10.1371/journal.pone.0144217)

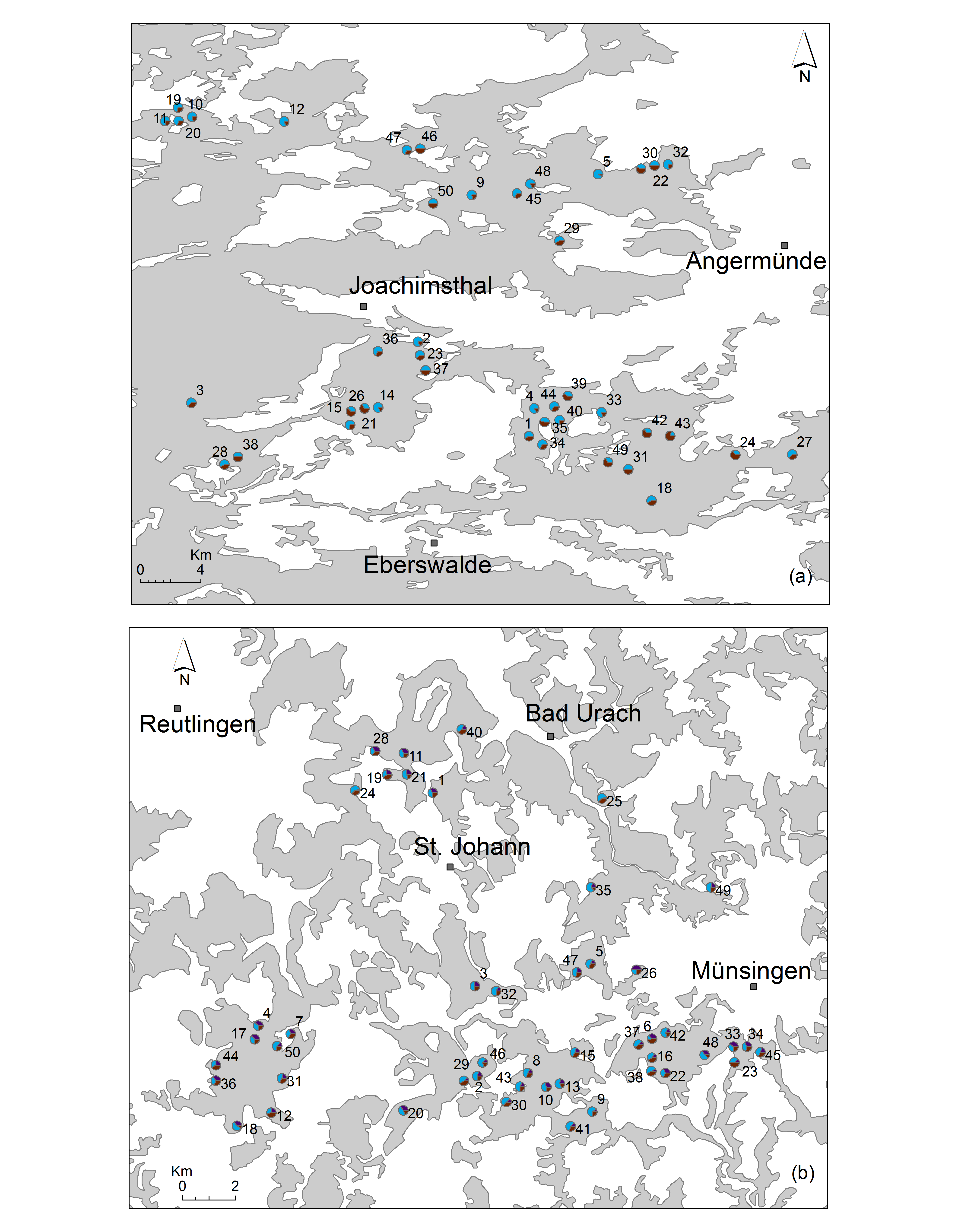

Supplement: S1 Fig — (a) Schorfheide-Chorin, (b) Schwäbische Alb. Individuals in the Schorfheide-Chorin were assigned to the cluster to which they had larger than a 50% chance of belonging as per STRUCTURE. The pie charts present the number of individuals sampled from each local population belonging to each of the clusters. Replacing the number of individuals belonging to each cluster with the likelihood of belonging to each cluster gives a similar pattern. Grey areas are forested (see legend of Fig 1). Squares indicate named towns and villages. Note that the scales of the maps are different. All maps were created using ArcGIS ver. 10.1 [43]. (TIF) [file pone.0144217.s001.tif]

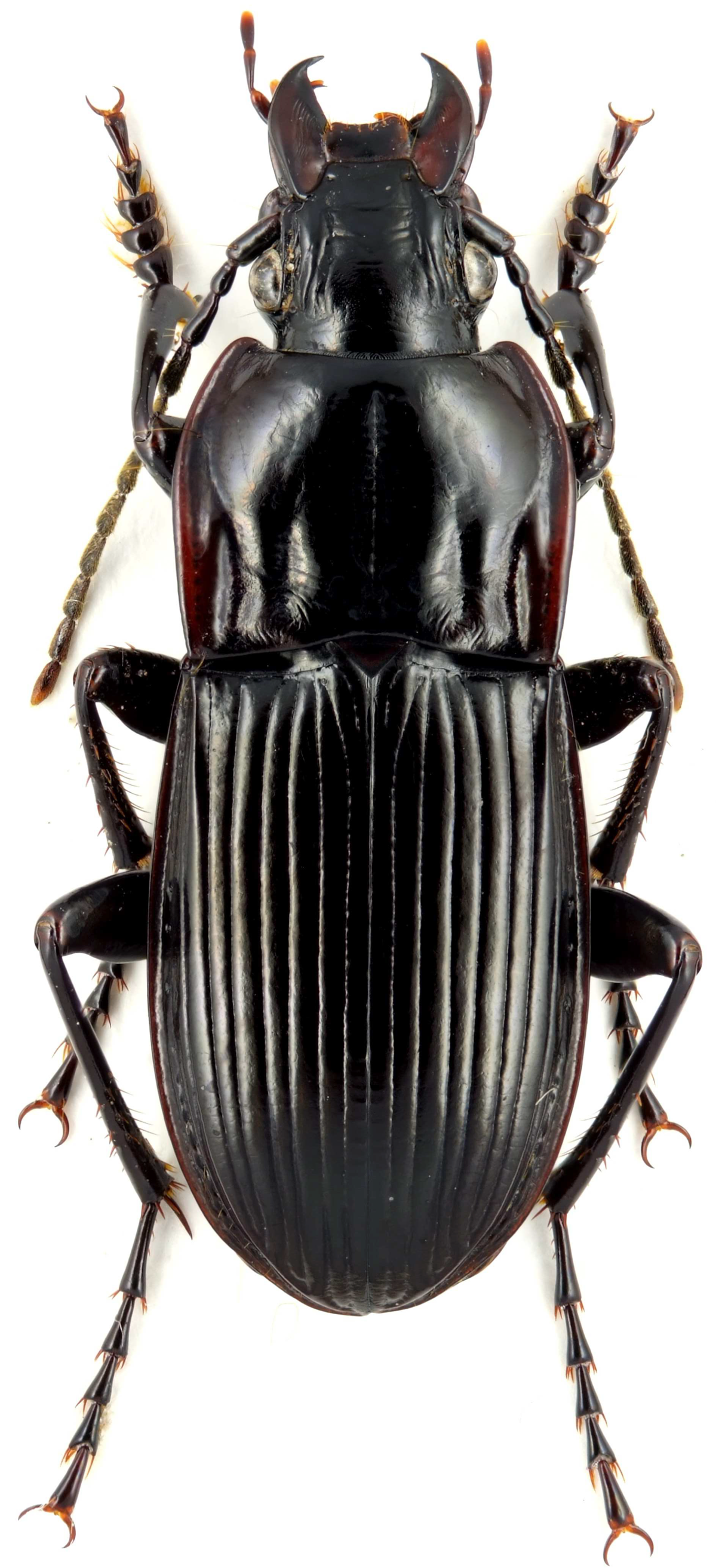

Supplement: S2 Fig — (TIF) [file pone.0144217.s002.tif]

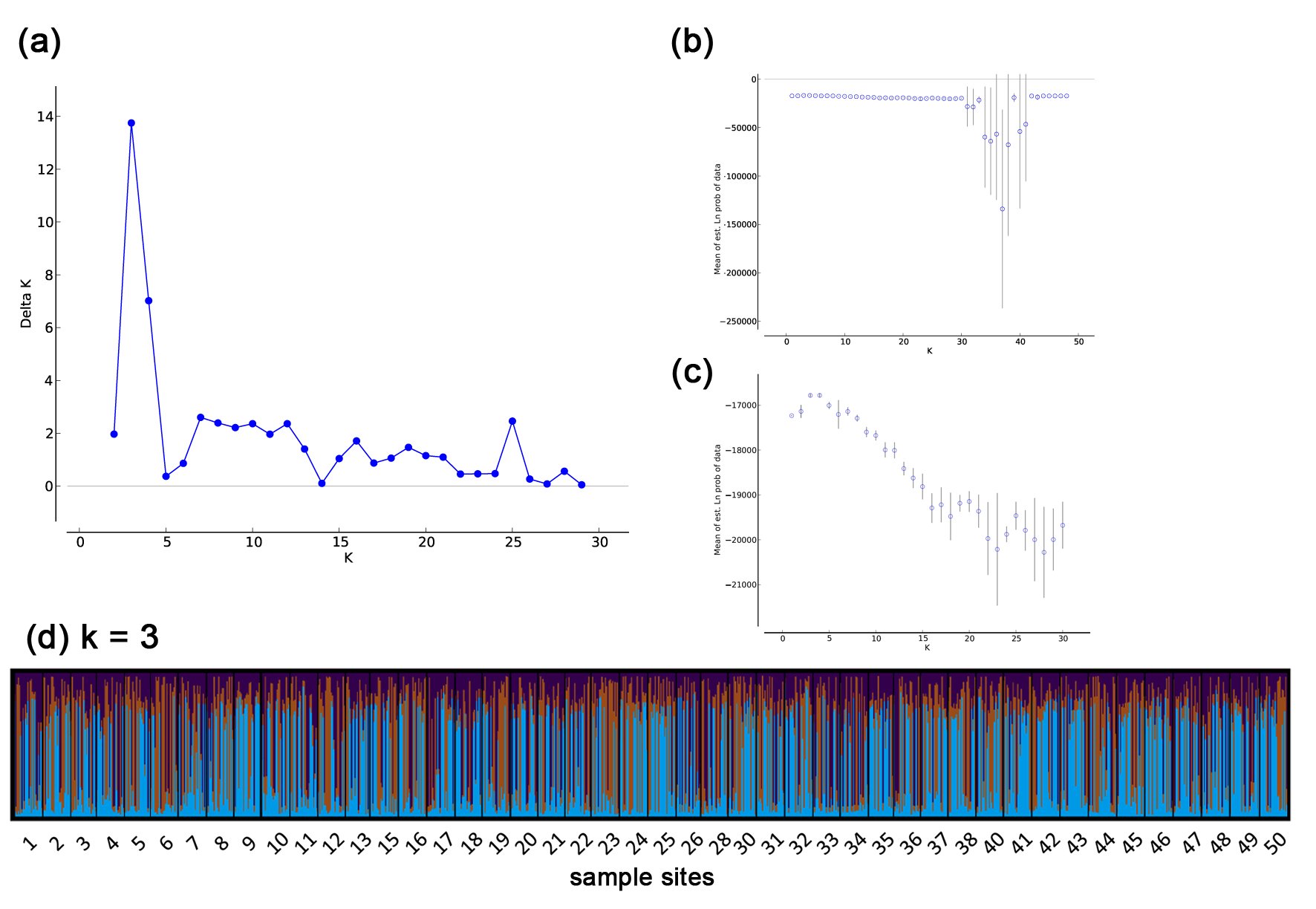

Supplement: S3 Fig — (a) ΔK/K plot, (b) mean likelihood and variance for each K–for K = 1 to K = 47, (c) mean likelihood and variance for each K–for K = 1 to K = 30, (d) membership probability of individuals for K = 3. (TIF) [file pone.0144217.s003.tif]

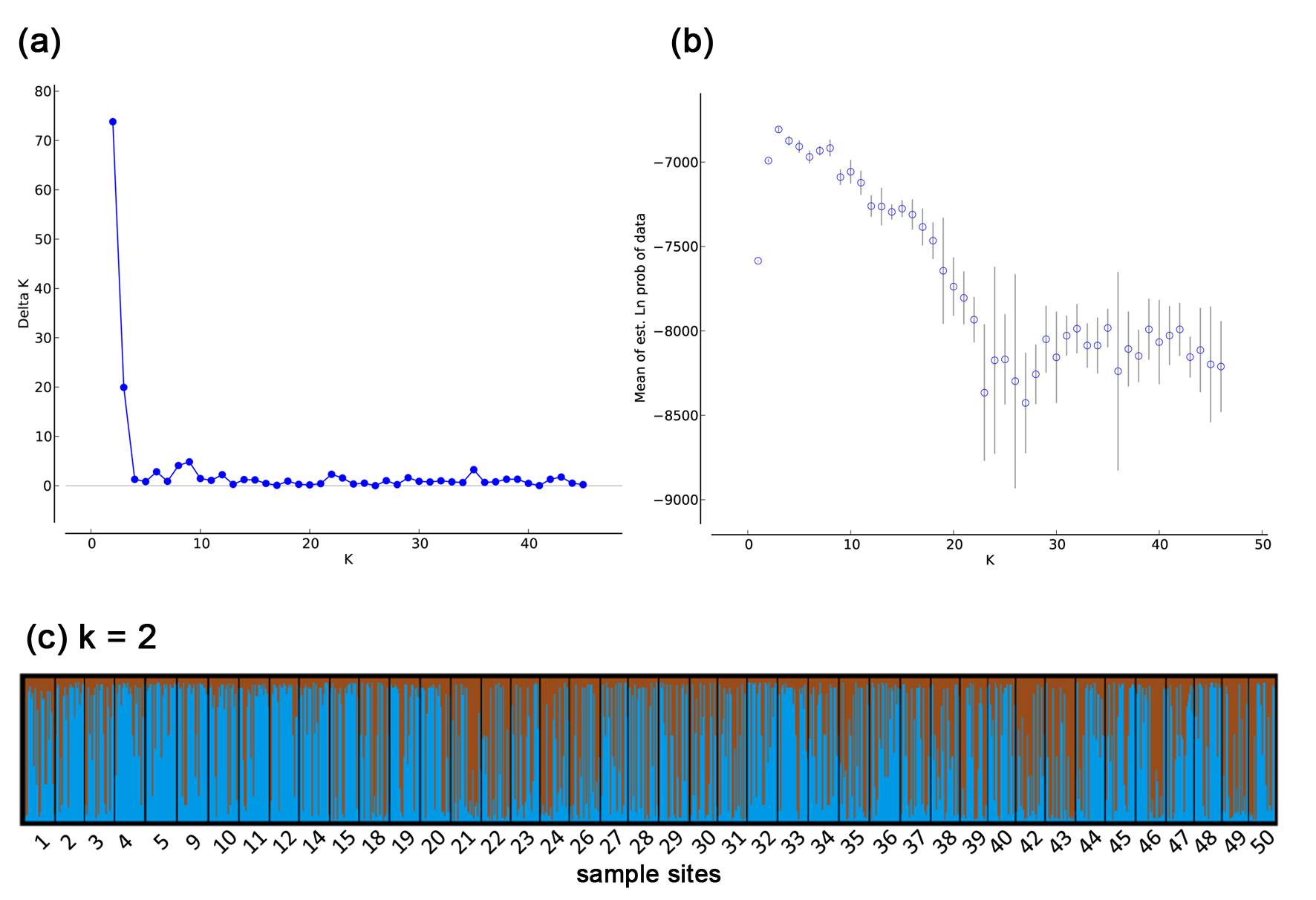

Supplement: S4 Fig — (a) ΔK/K plot, (b) mean likelihood and variance for each K–for K = 1 to K = 43, (c) membership probability of individuals for K = 2. (TIF) [file pone.0144217.s004.tif]

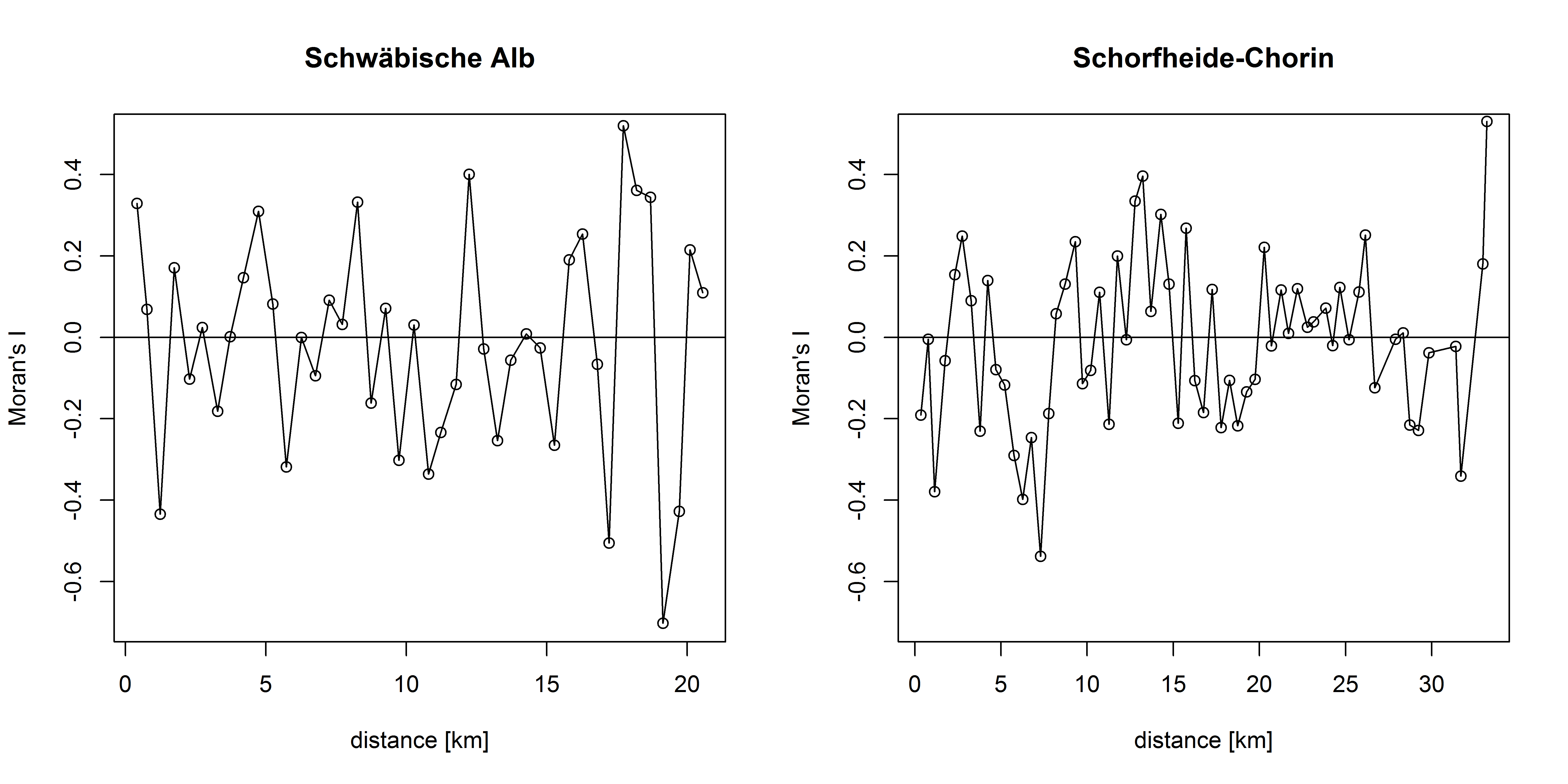

Supplement: S5 Fig — Empty circles indicate non-significant values. (TIF) [file pone.0144217.s005.tif]

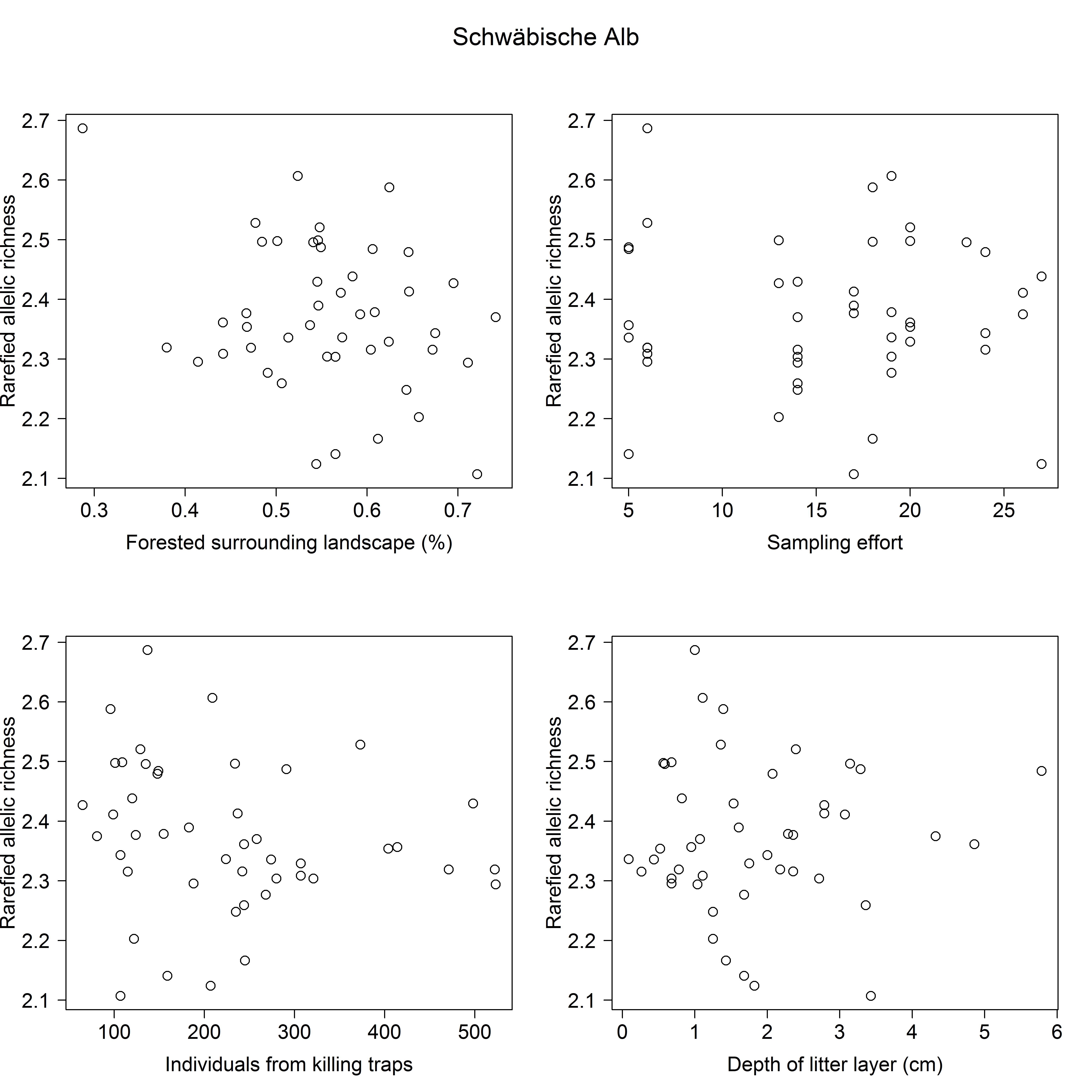

Supplement: S6 Fig — Relationship between rarefied allelic richness and proxies of population size and depth of the litter region for the Schwäbische Alb. Results are similar to those of the reported models (Spearman Rank Correlation; forested surrounding landscape: rho = -0.169, p = 0.261, sampling effort: rho = 0.088, p = 0.562, individuals from killing traps: rho = -0.286, p = 0.054, depth of litter layer: rho = 0.012, p = 0.935). (TIF) [file pone.0144217.s006.tif]

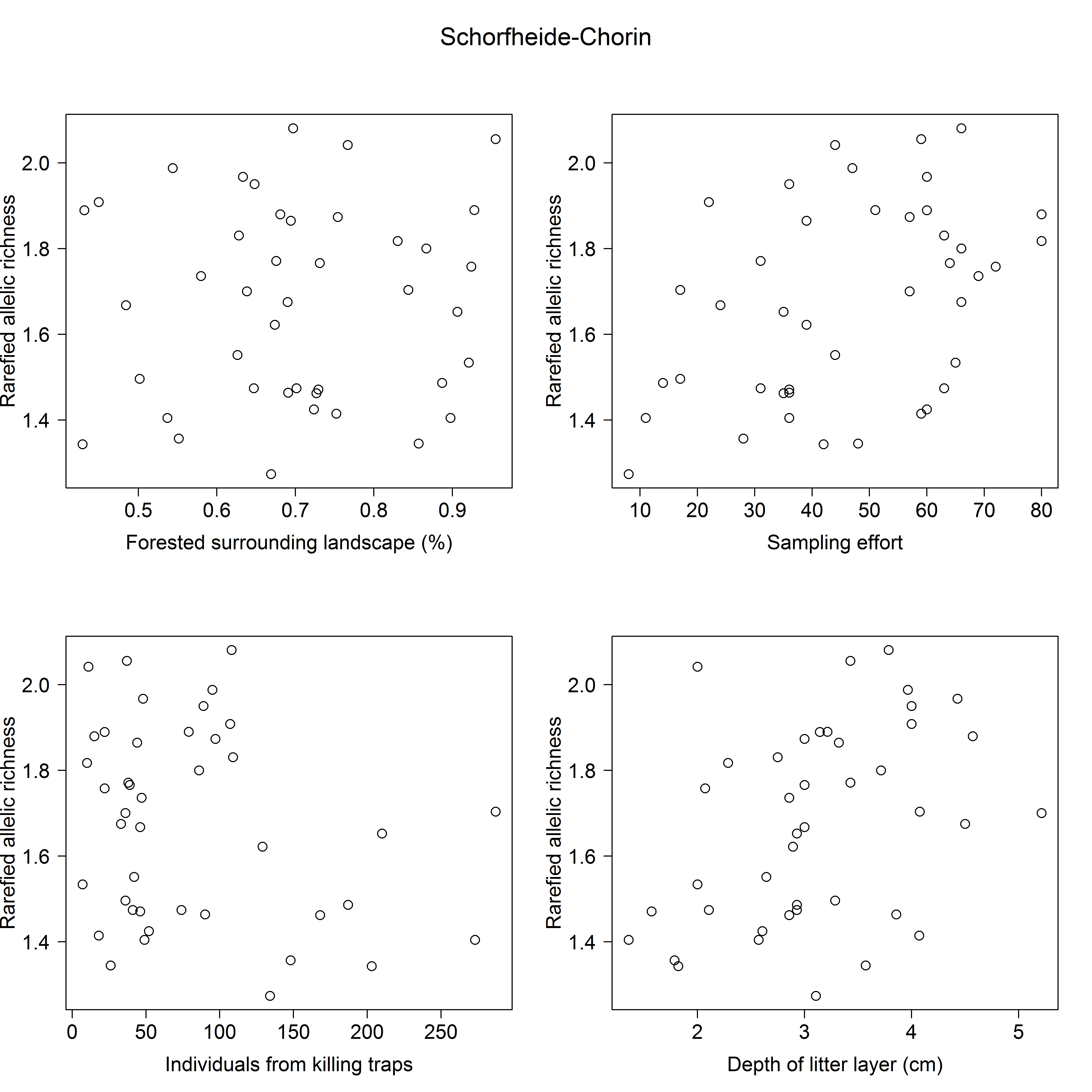

Supplement: S7 Fig — Relationship between rarefied allelic richness and proxies of population size and depth of the litter region for the Schorfheide-Chorin. Results are similar to those of the reported models (Spearman Rank Correlation; forested surrounding landscape: rho = 0.048, p = 0.761, sampling effort: rho = 0.397, p = 0.009, individuals from killing traps: rho = -0.212, p = 0.177, depth of litter layer: rho = 0.421, p = 0.005). (TIF) [file pone.0144217.s007.tif]
