# Supplementary material for: Living in Heterogeneous Woodlands – Are Habitat Continuity or Quality Drivers of Genetic Variability in a Flightless Ground Beetle?
Source: PLoS One. 2015 Dec 7;10(12):e0144217. doi: 10.1371/journal.pone.0144217 (PMC4671619; doi:10.1371/journal.pone.0144217)
Supplement: S1 Table — (PDF) [file pone.0144217.s008.pdf]

Table S1: Maps used to define woodlands as recent or ancient.

Region	Name	Year
Schwäbische Alb	Flurkartenwerk 1:2500 der Württembergischen Landesvermessung 1818-1840	1820
	Topographische Karte [Deutsches Reich]	1908-1912, 1930
	LANDSAT	1975
		1993
		2000
	CORINE Land cover	1990
		2000
		2006
	Automatische Liegenschaftskarte	2010
	Digitales Landschaftsmodell	2010
Schorfheide-Chorin	Schmettausches Kartenwerk	1767-1787
	Preußische Kartenaufnahme	1826
	Topographische Karte [Deutsches Reich]	1934-1943
	Physical surveys	2006
