# Supplementary material for: Living in Heterogeneous Woodlands – Are Habitat Continuity or Quality Drivers of Genetic Variability in a Flightless Ground Beetle?
Source: PLoS One. 2015 Dec 7;10(12):e0144217. doi: 10.1371/journal.pone.0144217 (PMC4671619; doi:10.1371/journal.pone.0144217)
Supplement: S2 Table — (PDF) [file pone.0144217.s009.pdf]

Table S2: Full list of variables used to characterize plots.

Variable	Explanation	Comment – citations
Allelic richness (AR)	rarefied number of alleles	Measure of genetic diversity. Response variable.
Map	Whether each plot is found in an ancient or in recent woodland.	see Table S1
Stand age	Age of trees on plot.	[1] Schwäbische Alb: Forsteinrichtung des Landes Baden-Württemberg, Regierungspräsidium Tübingen (2001-2003)<br>Schorfheide Chorin: Landesforstanstalt Eberswalde (2007)
Number of *Abax parallelepipedus* individuals	Collected using pitfall traps in 2008.	[2] Left out of model due to collinearity.
Sampling effort	Number of days needed to collect 33 *A. parallelepipedus* individuals from the plot.	
Forest management type		[3] Possible categories – age class forest, natural forest
Land use intensity	as per FORMI index	[4] Left out of model due to collinearity.
Percentage of surrounding landscape which is forested	Percentage of the buffer zone with a 2km radius surrounding the plot which is covered in trees.	As per the 2010 Digitales Landschaftsmodell maps (see supplementary methods)
Main tree species	Main tree species found on plot.	[3] Possible categories – European beech (*Fagus sylvatica*), sessile and pedunculated oak (*Quercus petraea* and *Quercus robur*), Scots pine (*Pinus sylvestris*), Norway spruce (*Picea abies*)
Cover of trees (>10m)	Percentage of plot covered by trees taller than 10 m.	For details see [5]
Cover of deadwood	Percentage of plot covered in deadwood	For details see [6]
Cover of litter	Percentage of plot which has a litter layer.	[2]
Number of vascular plant species		For details see [5]
Percentage of closed forest species	Percentage of the lichen, bryophyte, and vascular plant species which are restricted to closed forests.	For classification see [7]
Soil type		[3] Possible categories – albeluvisol, cambisol, leptosol, luvisol, podzol, regosol, stagnosol
Soil pH		[8] Left out of model due to collinearity.
CN ratio O_i soil layer	CN ratio of the litter layer. Included to reflect effects of historical land use on the soil.	[8]
CN ratio of O_e soil layer	CN ratio of the fermentation layer. Included to reflect effects of historical land use on the soil.	[8]
CN ratio of the A soil layer	CN ratio of the mineral horizon. Included to reflect effects of historical land use on the soil.	[8] Left out of model due to collinearity.
Depth of the O_i soil layer	Depth of the litter layer.	[8]
Depth of O_e soil layer	Depth of the fermentation horizon. Included to reflect effects of historical land use on the soil.	[8] Left out of model due to collinearity.
Depth of O_a soil layer	Depth of organic soil horizon.	[8]
Carbon content of the A horizon	Carbon content of the mineral horizon. Included to reflect effects of historical land use on the soil.	[8] Left out of model due to collinearity.
Latitude		[3] Left out of model due to collinearity.
Longitude		[3] Left out of model due to collinearity.
Elevation		[3] In meters above sea level
Mean annual precipitation		[9] Left out of model due to collinearity.
Annual mean temperature		Also represents precipitation as they are highly correlated. [9]

1. Schall P, Ammer C (2013) How to quantify forest management intensity in Central European forests. Eur J For Res 132: 379-396.
2. Lange M, Türke M, Pašalić E, Boch S, Hessenmöller D, et al. (2014) Effects of forest management on ground-dwelling beetles (Coleoptera; Carabidae, Staphylinidae) in Central Europe are mainly mediated by changes in forest structure. For Ecol Manage 329: 166-176.
3. Fischer M, Bossdorf O, Gockel S, Hänsel F, Hemp A, et al. (2010) Implementing large-scale and long-term functional biodiversity research: The Biodiversity Exploratories. Basic Appl Ecol 11: 473-485
4. Kahl T, Bauhus J (2014) An index of forest management intensity based on assessment of harvested tree volume, tree species composition and dead wood origin. Nat Conserv 7: 15-27.
5. Boch S, Prati D, Müller J, Socher SA, Baumbach H, Buscot F, Gockel S, Hemp A, Hessenmöller D, Kalko EKV, Linsenmair KE, Pfeiffer S, Pommer U, Schöning I, Schulze E-D, Seilwinder C, Weisser WW, Wells K, Fischer M (2013) High plant species richness indicates management-related disturbances, rather than the conservation status of forests. Basic Appl Ecol 14: 496–505.
6. Boch S, Prati D, Hessenmöller D, Schulze ED, Fischer M (2013) Richness of lichen species, especially of threatened ones, is promoted by management methods furthering stand continuity. PLoS ONE 8(1): e55461 doi: 10.1371/journal.pone.0055461.
7. Schmidt M, Kriebitzsch WU, Ewald J (2011) Waldartenlisten der Farn-und Blütenpflanzen, Moose und Flechten Deutschlands. Bonn: Bundesamt für Naturschutz. 111 p.
8. Solly EF, Schöning I, Boch S, Kandeler E, Marhan S, et al. (2014) Factors controlling decomposition rates of fine root litter in temperate forests and grasslands. Plant Soil 382: 203-218.

9. Hijmans RJ, Cameron SE, Parra JL, Jones PG, Jarvis A (2005) Very high resolution interpolated climate surfaces for global land areas. Int J Climatol 25: 1965-1978.
