# Supplementary material for: Living in Heterogeneous Woodlands – Are Habitat Continuity or Quality Drivers of Genetic Variability in a Flightless Ground Beetle?
Source: PLoS One. 2015 Dec 7;10(12):e0144217. doi: 10.1371/journal.pone.0144217 (PMC4671619; doi:10.1371/journal.pone.0144217)
Supplement: S3 Table — Values greater than |0.7| are marked in bold. (PDF) [file pone.0144217.s010.pdf]

Table S3: Collinearity matrix of continuous predictor variables for variable selection (Spearman's rho values). Values greater than |0.7| are marked in bold.

	Allelic richness	Soil pH	CN ratio Oi soil layer	CN ratio of Oe soil layer	CN ratio of the A soil layer	Annual mean temperature	Mean annual precipitation	Sampling effort	Cover of trees (>10m)	Cover of litter	Cover of deadwood	Number of vascular plant species	Percentage of surrounding landscape which is forested	Depth of the Oi soil layer	Depth of Oe soil layer	Depth of Oa soil layer	Percentage of closed forest species	Sampling effort	Longitude	Latitude	Elevation	Carbon content of the A horizon	Stand age	Land use intensity
Allelic richness	1																							
Soil pH	0.66	1																						
CN ratio Oi soil layer	-0.3	-0.2	1																					
CN ratio of Oe soil layer	**-0.76**	**-0.7**	0.51	1																				
CN ratio of the A soil layer	**-0.78**	**-0.84**	0.32	**0.76**	1																			
Annual mean temperature	**-0.73**	**-0.73**	0.15	0.64	**0.78**	1																		
Mean annual precipitation	**0.72**	0.7	-0.19	**-0.71**	**-0.81**	**-0.93**	1																	
Sampling effort	0.47	0.65	-0.12	-0.42	-0.56	-0.65	0.6	1																
Cover of trees (>10m)	0.03	0.1	0.02	0.03	-0.12	-0.06	0.06	0.19	1															
Cover of litter	0.08	0.2	-0.09	-0.01	-0.25	-0.09	0.05	0.25	0.5	1														
Cover of deadwood	-0.01	-0.09	0.05	-0.05	0.14	-0.05	0.04	0.01	-0.32	-0.48	1													
Number of vascular plant species	0.63	0.5	-0.19	-0.61	-0.54	-0.53	0.53	0.26	-0.46	-0.35	0.17	1												
Percentage of surrounding landscape which is forested	-0.47	-0.42	-0.04	0.3	0.37	0.47	-0.36	-0.48	0.05	-0.04	-0.06	-0.4	1											
Depth of the Oi soil layer	-0.39	-0.34	-0.02	0.39	0.34	0.52	-0.54	-0.47	0.17	0.31	-0.38	-0.47	0.34	1										
Depth of Oe soil layer	-0.64	**-0.7**	0.25	0.64	**0.78**	**0.73**	**-0.76**	-0.63	-0.09	0.01	-0.11	-0.53	0.4	0.65	1									
Depth of Oa soil layer	-0.55	-0.52	0.3	0.59	0.67	0.49	-0.58	-0.33	-0.14	0.02	0.12	-0.45	0.16	0.17	0.59	1								
Percentage of closed forest species	0.41	0.45	-0.19	-0.33	-0.56	-0.42	0.37	0.41	0.4	0.42	-0.32	0.08	-0.13	-0.03	-0.43	-0.32	1							
Sampling effort	-0.54	**-0.71**	0.14	0.53	0.65	0.68	-0.65	**-0.75**	-0.08	-0.21	-0.02	-0.32	0.5	0.43	0.63	0.31	-0.34	1						
Longitude	**-0.72**	-0.67	0.22	**0.74**	**0.76**	**0.73**	**-0.86**	-0.51	0.07	0.01	-0.03	-0.59	0.34	0.56	**0.72**	0.58	-0.2	0.62	1					
Latitude	**-0.74**	**-0.75**	0.31	**0.8**	**0.77**	0.65	**-0.73**	-0.53	0.01	-0.15	-0.06	-0.64	0.31	0.4	0.69	0.58	-0.37	0.57	0.7	1				
Elevation	**0.75**	**0.75**	-0.17	-0.68	**-0.83**	**-0.93**	**0.93**	0.62	0.16	0.18	-0.02	0.45	-0.36	-0.47	**-0.73**	-0.52	0.48	-0.64	**-0.75**	**-0.72**	1			
Carbon content of the A horizon	**0.75**	**0.72**	-0.19	**-0.73**	**-0.74**	**-0.77**	**0.77**	0.63	-0.01	0.03	0.08	0.59	-0.44	-0.46	-0.69	-0.51	0.29	-0.62	**-0.74**	**-0.73**	**0.75**	1		
Stand age	-0.07	0.01	-0.13	0.17	-0.08	0.07	-0.07	0.04	0.26	0.44	-0.46	-0.32	0.13	0.46	0.12	0.01	0.36	0.06	0.17	0.04	0	-0.14	1	
Land use intensity	0.06	-0.07	0.05	-0.15	0.15	-0.05	0.04	-0.09	-0.48	-0.53	0.49	0.39	-0.17	-0.36	-0.04	0.01	-0.46	-0.03	-0.16	-0.07	-0.08	0.15	**-0.76**	1
