# Supplementary material for: Living in Heterogeneous Woodlands – Are Habitat Continuity or Quality Drivers of Genetic Variability in a Flightless Ground Beetle?
Source: PLoS One. 2015 Dec 7;10(12):e0144217. doi: 10.1371/journal.pone.0144217 (PMC4671619; doi:10.1371/journal.pone.0144217)
Supplement: S4 Table — (PDF) [file pone.0144217.s011.pdf]

Table S4: Allele frequencies and related indices.

Plot	Alb1	Alb10	Alb11	Alb12	Alb13	Alb15	Alb16	Alb17	Alb18	Alb19	Alb2	Alb20	Alb21	Alb22	Alb23	Alb24	Alb25	Alb26	Alb28	Alb3	Alb30	Alb31	Alb32	Alb33	Alb34	Alb35	Alb36	Alb37	Alb38	Alb4	Alb40	Alb41	Alb42	Alb43	Alb44	Alb45	Alb46	Alb47	Alb48	Alb49	Alb5	Alb50	Alb6	Alb7	Alb8	Alb9	Sch1	Sch10	Sch11	Sch12	Sch14	Sch15	Sch18	Sch19	Sch2	Sch20	Sch21	Sch22	Sch23	Sch24	Sch26	Sch27	Sch28	Sch29	Sch3	Sch30	Sch31	Sch32	Sch33	Sch34	Sch35	Sch36	Sch37	Sch38	Sch39	Sch4	Sch40	Sch42	Sch43	Sch44	Sch45	Sch46	Sch47	Sch48	Sch49	Sch5	Sch50	Sch9
Locus 2																																																																																										
165	-	-	-	-	-	-	-	-	-	-	-	-	-	-	-	-	-	-	-	-	-	-	-	-	-	-	-	-	-	-	-	-	-	-	-	-	-	-	-	-	-	-	-	-	-	-	-	-	-	-	-	0.023	-	-	-	-	-	-	-	-	-	-	-	-	-	-	-	-	-	0.021	-	-	-	-	-	-	-	-	-	-	-	-	-	-	-	-	-	-
167	-	-	-	-	-	-	-	-	-	-	0.021	-	-	-	-	-	-	0.024	-	-	-	-	-	-	-	-	-	-	-	-	-	-	-	-	-	-	-	-	-	-	-	-	-	-	-	-	-	-	-	-	-	-	-	-	-	-	-	-	-	-	-	-	-	-	-	-	-	-	-	-	-	-	-	-	-	-	-	-	-	-	-	-	-	-	-	-	-	-
169	0.083	0.229	0.271	0.217	0.262	0.417	0.333	0.208	0.152	0.188	0.167	0.196	0.283	0.341	0.146	0.229	0.208	0.214	0.174	0.25	0.125	0.083	0.313	0.261	0.188	0.19	0.283	0.283	0.271	0.125	0.174	0.229	0.417	0.25	0.188	0.292	0.25	0.25	0.208	0.271	0.261	0.229	0.208	0.208	0.261	0.25	-	-	-	-	-	0.023	0.042	-	-	0.063	-	0.065	0.065	0.022	0.083	0.023	0.063	0.063	-	0.114	0.022	-	-	-	0.021	0.021	-	0.043	0.068	-	0.023	0.065	0.104	0.043	-	0.104	0.045	-	-	-	0.068	-
171	0.896	0.771	0.729	0.783	0.738	0.583	0.667	0.792	0.848	0.792	0.813	0.804	0.717	0.659	0.854	0.771	0.771	0.738	0.804	0.75	0.854	0.896	0.688	0.739	0.813	0.81	0.717	0.717	0.729	0.875	0.826	0.771	0.583	0.729	0.813	0.708	0.75	0.75	0.792	0.729	0.739	0.771	0.75	0.792	0.739	0.727	1	1	1	0.978	1	0.955	0.958	1	1	0.938	1	0.935	0.935	0.978	0.917	0.977	0.938	0.938	1	0.886	0.978	1	1	0.979	0.958	0.979	1	0.957	0.932	1	0.977	0.935	0.896	0.957	1	0.896	0.955	1	1	1	0.932	1
173	0.021	-	-	-	-	-	-	-	-	0.021	-	-	-	-	-	-	0.021	0.024	0.022	-	0.021	0.021	-	-	-	-	-	-	-	-	-	-	-	-	-	-	-	-	-	-	-	-	0.042	-	-	0.023	-	-	-	0.022	-	-	-	-	-	-	-	-	-	-	-	-	-	-	-	-	-	-	-	-	0.021	-	-	-	-	-	-	-	-	-	-	-	-	-	-	-	-	-
175	-	-	-	-	-	-	-	-	-	-	-	-	-	-	-	-	-	-	-	-	-	-	-	-	-	-	-	-	-	-	-	-	-	0.021	-	-	-	-	-	-	-	-	-	-	-	-	-	-	-	-	-	-	-	-	-	-	-	-	-	-	-	-	-	-	-	-	-	-	-	-	-	-	-	-	-	-	-	-	-	-	-	-	-	-	-	-	-	-
Locus 5																																																																																										
149	-	-	-	-	-	-	-	-	-	-	-	-	-	-	-	-	-	-	-	-	-	-	-	-	-	0.024	-	-	-	-	-	-	-	-	-	-	-	-	-	-	-	-	-	-	-	-	-	-	-	-	-	-	-	-	-	-	-	-	-	-	-	-	-	-	-	-	-	-	-	-	-	-	-	-	0.023	-	-	-	-	-	-	-	-	-	-	-	-	-
151	-	0.021	-	-	-	0.042	0.021	-	0.043	-	-	-	-	-	-	-	-	0.024	-	-	-	-	-	-	-	-	-	-	-	-	-	-	-	-	-	-	-	-	-	-	-	-	0.021	-	-	-	-	-	-	-	-	0.023	-	-	-	-	-	-	-	-	-	-	-	-	-	-	-	-	-	-	-	-	-	-	-	-	-	-	-	-	-	-	-	-	-	-	-	-
153	0.792	0.771	0.729	0.783	0.833	0.729	0.813	0.854	0.652	0.875	0.804	0.783	0.761	0.795	0.792	0.771	0.667	0.762	0.804	0.864	0.854	0.688	0.813	0.761	0.75	0.762	0.826	0.891	0.729	0.896	0.826	0.813	0.813	0.75	0.875	0.875	0.917	0.854	0.833	0.75	0.804	0.75	0.792	0.875	0.717	0.886	0.563	0.813	0.646	0.543	0.479	0.477	0.375	0.604	0.435	0.792	0.604	0.478	0.391	0.63	0.688	0.295	0.604	0.521	0.438	0.409	0.63	0.188	0.688	0.521	0.563	0.542	0.583	0.739	0.614	0.542	0.545	0.587	0.771	0.63	0.542	0.667	0.545	0.409	0.429	0.208	0.364	0.292
155	0.021	-	-	-	-	-	-	-	-	-	-	-	-	-	-	-	-	0.024	-	-	-	-	-	-	-	-	-	-	-	-	-	-	-	-	-	-	-	-	-	-	-	-	-	-	-	-	-	-	-	-	-	-	-	-	-	-	-	-	-	-	-	-	-	-	-	-	-	-	-	-	-	-	-	-	-	-	-	-	-	-	-	-	-	-	-	-	-	-
157	0.188	0.208	0.271	0.217	0.167	0.229	0.167	0.146	0.304	0.125	0.196	0.217	0.239	0.205	0.208	0.229	0.333	0.19	0.196	0.136	0.146	0.313	0.188	0.239	0.25	0.214	0.174	0.109	0.271	0.104	0.174	0.188	0.188	0.25	0.125	0.125	0.083	0.146	0.167	0.25	0.196	0.25	0.188	0.125	0.283	0.114	0.438	0.188	0.354	0.457	0.521	0.5	0.625	0.396	0.565	0.208	0.396	0.522	0.609	0.37	0.313	0.705	0.396	0.479	0.563	0.591	0.37	0.813	0.313	0.479	0.438	0.458	0.396	0.261	0.364	0.458	0.455	0.413	0.229	0.37	0.458	0.333	0.455	0.591	0.571	0.792	0.636	0.708
159	-	-	-	-	-	-	-	-	-	-	-	-	-	-	-	-	-	-	-	-	-	-	-	-	-	-	-	-	-	-	-	-	-	-	-	-	-	-	-	-	-	-	-	-	-	-	-	-	-	-	-	-	-	-	-	-	-	-	-	-	-	-	-	-	-	-	-	-	-	-	-	-	0.021	-	-	-	-	-	-	-	-	-	-	-	-	-	-	-
Locus 6																																																																																										
286	0.104	0.021	0.063	0.174	0.143	0.042	0.042	0.042	0.087	0.063	0.063	0.109	0.087	0.045	0.146	0.167	0.083	0.048	0.065	0.114	0.083	0.063	0.063	0.065	0.125	0.024	0.205	0.109	0.125	0.167	0.109	0.167	0.104	0.125	0.083	0.104	0.021	0.104	0.063	0.021	0.109	0.174	0.063	-	0.174	0.114	-	-	-	-	-	-	-	-	-	-	-	0.043	-	0.022	0.042	-	0.042	0.043	-	0.068	0.022	0.042	-	-	-	0.042	-	-	0.023	-	0.023	0.022	0.063	0.022	-	0.083	0.023	-	0.024	-	0.068	-
288	0.833	0.979	0.938	0.826	0.857	0.958	0.958	0.958	0.913	0.938	0.938	0.891	0.913	0.955	0.854	0.833	0.917	0.952	0.935	0.886	0.917	0.938	0.938	0.935	0.875	0.976	0.795	0.891	0.854	0.833	0.891	0.833	0.896	0.875	0.917	0.896	0.979	0.896	0.938	0.979	0.891	0.826	0.938	1	0.826	0.886	1	1	1	1	1	1	1	1	1	1	1	0.957	1	0.978	0.958	1	0.958	0.957	1	0.932	0.978	0.958	1	1	1	0.958	1	1	0.977	1	0.977	0.978	0.938	0.978	1	0.917	0.977	1	0.976	1	0.932	1
290	0.063	-	-	-	-	-	-	-	-	-	-	-	-	-	-	-	-	-	-	-	-	-	-	-	-	-	-	-	0.021	-	-	-	-	-	-	-	-	-	-	-	-	-	-	-	-	-	-	-	-	-	-	-	-	-	-	-	-	-	-	-	-	-	-	-	-	-	-	-	-	-	-	-	-	-	-	-	-	-	-	-	-	-	-	-	-	-	-	-
Locus12																																																																																										
124	-	-	-	-	-	-	-	-	-	-	-	-	-	-	-	-	-	-	-	-	-	-	-	-	-	-	-	-	-	-	-	-	-	-	-	-	-	-	-	-	-	-	-	-	-	-	-	-	-	-	-	-	-	-	-	0.021	-	-	-	-	-	-	-	-	-	-	-	-	-	-	-	-	-	-	-	-	-	-	-	-	-	-	-	-	-	-	-	-
126	0.042	-	-	0.043	0.071	0.083	-	0.022	0.043	0.021	-	0.022	0.022	0.091	-	-	-	-	-	-	-	0.021	0.021	0.022	0.021	0.048	0.043	0.065	-	-	0.087	0.042	0.021	-	-	-	-	-	-	-	-	0.021	-	-	-	-	-	-	-	-	-	-	-	-	-	-	-	-	-	-	-	-	-	-	-	-	-	-	-	-	-	-	-	-	-	-	-	-	-	-	-	-	-	-	-	-	-	-
128	-	-	-	-	-	-	-	-	-	-	-	-	-	-	-	-	-	-	-	-	-	-	-	-	-	-	-	-	-	-	-	-	0.021	-	-	-	-	-	-	-	-	-	-	-	-	-	-	-	-	-	-	-	0.021	-	-	-	-	-	-	-	-	-	-	-	-	-	-	-	-	-	-	-	-	-	-	-	-	-	-	-	-	-	-	-	-	-	-	-
132	-	-	-	-	-	-	-	-	-	-	-	-	-	-	-	-	-	-	-	-	-	-	-	-	-	-	-	-	-	-	-	-	-	-	0.043	-	-	-	-	-	-	-	-	-	-	-	-	-	-	-	-	-	-	-	-	-	-	-	-	-	-	-	-	-	-	-	-	-	-	-	-	-	-	-	-	-	-	-	0.021	-	-	-	-	-	-	-	-	-
134	0.938	0.979	1	0.891	0.905	0.896	0.979	0.978	0.935	0.917	0.979	0.935	0.957	0.886	0.979	0.917	0.938	0.95	0.978	0.932	0.979	0.896	0.896	0.978	0.958	0.905	0.891	0.891	0.979	0.979	0.891	0.938	0.917	1	0.87	1	0.979	0.938	1	0.891	0.935	0.938	0.979	0.938	0.978	1	0.979	1	1	0.978	1	0.932	0.958	1	0.978	0.979	0.958	0.957	1	1	1	1	0.896	1	1	1	1	1	0.958	1	1	0.958	0.958	0.957	0.909	1	0.977	1	0.958	0.978	1	0.979	1	1	0.952	0.979	1	0.979
136	0.021	0.021	-	0.065	0.024	0.021	0.021	-	0.022	0.063	0.021	0.022	0.022	0.023	0.021	0.083	0.042	0.025	0.022	0.068	0.021	0.063	0.042	-	0.021	0.048	0.065	0.043	0.021	-	0.022	0.021	0.021	-	0.087	-	0.021	0.063	-	0.043	0.065	0.042	0.021	0.063	0.022	-	0.021	-	-	0.022	-	0.068	0.021	-	0.022	-	0.021	0.043	-	-	-	-	0.063	-	-	-	-	-	0.042	-	-	-	-	0.022	0.091	-	0.023	-	0.021	0.022	-	0.021	-	-	0.048	0.021	-	0.021
138	-	-	-	-	-	-	-	-	-	-	-	0.022	-	-	-	-	-	0.025	-	-	-	0.021	0.042	-	-	-	-	-	-	0.021	-	-	0.021	-	-	-	-	-	-	0.065	-	-	-	-	-	-	-	-	-	-	-	-	-	-	-	-	0.021	-	-	-	-	-	0.042	-	-	-	-	-	-	-	-	0.042	0.042	0.022	-	-	-	-	-	-	-	-	-	-	-	-	-	-
140	-	-	-	-	-	-	-	-	-	-	-	-	-	-	-	-	0.021	-	-	-	-	-	-	-	-	-	-	-	-	-	-	-	-	-	-	-	-	-	-	-	-	-	-	-	-	-	-	-	-	-	-	-	-	-	-	-	-	-	-	-	-	-	-	-	-	-	-	-	-	-	-	-	-	-	-	-	-	-	-	-	-	-	-	-	-	-	-	-
Locus 14																																																																																										
112	-	0.021	0.042	-	-	0.021	0.021	0.042	0.065	0.021	-	-	-	-	-	-	-	-	-	-	-	-	-	-	-	-	0.022	-	-	0.021	-	-	-	-	0.021	-	-	-	-	-	-	-	0.063	0.021	-	-	-	-	-	-	0.021	0.045	-	-	-	-	-	-	-	-	-	-	-	-	0.021	-	-	-	-	-	-	-	-	-	-	-	-	-	0.021	-	-	-	-	-	-	-	-	-
116	-	0.021	-	-	-	0.021	-	-	-	-	-	-	-	-	-	-	-	-	-	-	-	-	-	-	-	-	-	-	-	-	-	-	-	-	0.021	-	-	-	-	-	0.023	-	-	-	-	-	-	-	-	-	-	-	-	-	-	-	-	-	-	-	-	-	-	-	-	-	-	-	-	-	-	-	-	-	-	-	-	-	-	-	-	-	-	-	-	-	-	-
118	-	-	-	-	-	-	-	-	-	-	-	-	-	-	-	-	-	-	-	-	-	-	-	-	-	-	-	-	-	-	-	-	-	-	-	-	-	-	-	-	-	-	-	-	-	-	-	-	-	-	-	-	-	-	-	-	-	-	-	-	-	-	-	-	-	-	-	-	-	0.021	-	-	-	-	-	-	-	-	-	-	-	-	-	-	-	-	-	-
120	0.313	0.125	0.25	0.261	0.143	0.271	0.271	0.083	0.109	0.333	0.375	0.109	0.37	0.273	0.271	0.146	0.188	0.167	0.326	0.273	0.208	0.167	0.271	0.261	0.292	0.31	0.217	0.239	0.208	0.292	0.261	0.208	0.208	0.208	0.188	0.208	0.25	0.25	0.396	0.354	0.295	0.188	0.25	0.167	0.283	0.114	0.042	0.021	-	-	0.021	0.045	0.063	0.063	0.065	0.083	0.354	0.261	0.114	0.239	0.104	0.045	-	0.167	-	0.068	0.13	-	0.042	0.063	0.208	0.188	0.104	0.174	0.182	0.021	0.136	0.065	0.25	0.109	0.063	0.188	0.114	0.068	0.143	0.021	0.136	-
122	0.688	0.813	0.688	0.739	0.833	0.688	0.708	0.875	0.783	0.625	0.604	0.891	0.609	0.682	0.729	0.854	0.771	0.833	0.674	0.705	0.792	0.833	0.708	0.739	0.708	0.69	0.761	0.739	0.792	0.688	0.739	0.792	0.771	0.792	0.771	0.75	0.75	0.75	0.604	0.583	0.682	0.792	0.688	0.792	0.674	0.864	0.958	0.979	1	1	0.958	0.909	0.938	0.938	0.935	0.917	0.646	0.717	0.886	0.761	0.896	0.955	1	0.813	0.979	0.932	0.87	0.979	0.854	0.917	0.792	0.813	0.896	0.826	0.818	0.979	0.864	0.935	0.729	0.891	0.938	0.813	0.886	0.932	0.857	0.979	0.864	1
124	-	0.021	0.021	-	0.024	-	-	-	0.043	0.021	0.021	-	0.022	0.045	-	-	0.042	-	-	0.023	-	-	0.021	-	-	-	-	0.022	-	-	-	-	0.021	-	-	0.042	-	-	-	0.063	-	0.021	-	0.021	0.043	0.023	-	-	-	-	-	-	-	-	-	-	-	0.022	-	-	-	-	-	0.021	-	-	-	0.021	0.104	-	-	-	-	-	-	-	-	-	-	-	-	-	-	-	-	-	-	-
Locus 20																																																																																										
186	0.354	0.396	0.375	0.565	0.571	0.313	0.438	0.396	0.432	0.458	0.333	0.457	0.261	0.5	0.292	0.458	0.313	0.429	0.5	0.432	0.583	0.458	0.583	0.478	0.375	0.429	0.5	0.478	0.396	0.313	0.37	0.479	0.521	0.5	0.375	0.521	0.354	0.375	0.521	0.417	0.587	0.542	0.375	0.583	0.348	0.545	-	-	-	-	-	0.023	0.083	-	-	-	0.063	0.109	0.043	0.022	-	-	-	-	-	0.023	0.065	-	-	-	0.042	-	-	0.022	0.068	-	-	0.043	0.063	-	0.021	0.063	0.023	-	-	-	0.023	-
188	0.646	0.604	0.625	0.435	0.429	0.688	0.563	0.604	0.568	0.542	0.667	0.543	0.717	0.5	0.708	0.542	0.688	0.548	0.5	0.568	0.417	0.542	0.417	0.522	0.625	0.571	0.5	0.522	0.604	0.688	0.63	0.521	0.479	0.5	0.625	0.479	0.646	0.625	0.458	0.583	0.413	0.458	0.625	0.396	0.652	0.455	1	1	1	1	1	0.977	0.917	1	1	1	0.938	0.891	0.957	0.978	1	1	1	1	1	0.977	0.935	1	1	1	0.958	1	1	0.978	0.932	1	1	0.957	0.938	1	0.979	0.938	0.977	1	1	1	0.977	1
190	-	-	-	-	-	-	-	-	-	-	-	-	0.022	-	-	-	-	0.024	-	-	-	-	-	-	-	-	-	-	-	-	-	-	-	-	-	-	-	-	0.021	-	-	-	-	0.021	-	-	-	-	-	-	-	-	-	-	-	-	-	-	-	-	-	-	-	-	-	-	-	-	-	-	-	-	-	-	-	-	-	-	-	-	-	-	-	-	-	-	-	-
Locus 23																																																																																										
161	-	-	-	-	-	-	-	-	-	-	-	-	-	-	-	-	-	-	-	-	-	-	-	-	-	-	-	-	-	-	-	-	-	-	-	-	-	0.021	-	-	-	-	-	-	-	-	-	-	-	-	-	-	-	-	-	-	-	-	-	-	-	-	-	-	-	-	-	-	-	-	-	-	-	-	-	-	-	-	-	-	-	-	-	-	-	-	-	-
163	-	-	0.022	-	-	-	-	-	-	-	-	-	-	-	-	-	-	-	-	-	0.021	-	0.125	-	-	-	-	-	-	-	-	-	-	-	-	-	-	-	-	-	-	-	-	-	-	-	-	-	-	-	-	-	-	-	-	-	-	-	-	-	-	-	-	-	-	-	-	-	-	-	-	-	-	-	-	-	-	-	-	-	-	-	-	-	-	-	-	-
165	1	1	0.957	1	1	1	0.979	1	1	1	0.938	1	0.957	0.977	1	1	1	1	0.935	0.977	0.958	1	0.875	1	0.979	0.976	1	1	1	1	0.978	0.979	0.979	0.979	1	1	0.979	0.958	1	0.979	1	1	1	0.978	1	1	1	0.979	0.938	0.978	1	0.957	0.979	0.958	0.935	0.958	1	1	0.957	1	1	1	1	1	0.979	0.977	0.957	1	1	1	1	0.979	1	1	0.977	1	1	1	1	1	1	0.979	1	1	0.976	1	1	1
167	-	-	0.022	-	-	-	0.021	-	-	-	0.063	-	0.043	0.023	-	-	-	-	0.065	0.023	0.021	-	-	-	0.021	0.024	-	-	-	-	0.022	0.021	0.021	0.021	-	-	0.021	0.021	-	0.021	-	-	-	0.022	-	-	-	0.021	0.063	0.022	-	0.043	0.021	0.042	0.065	0.042	-	-	0.043	-	-	-	-	-	0.021	0.023	0.043	-	-	-	-	0.021	-	-	0.023	-	-	-	-	-	-	0.021	-	-	0.024	-	-	-
Locus 25																																																																																										
203	0.479	0.375	0.5	0.435	0.452	0.458	0.458	0.478	0.522	0.563	0.458	0.609	0.457	0.432	0.542	0.417	0.5	0.525	0.636	0.386	0.417	0.479	0.5	0.391	0.396	0.357	0.522	0.5	0.583	0.479	0.565	0.5	0.542	0.417	0.438	0.5	0.479	0.604	0.432	0.438	0.409	0.5	0.417	0.375	0.543	0.455	1	1	1	1	1	0.886	0.896	1	1	1	0.917	0.886	0.957	1	0.979	1	1	0.958	1	0.977	0.913	1	1	1	0.938	0.979	1	0.957	0.955	1	0.955	0.957	0.938	1	0.979	0.958	0.955	1	1	1	0.977	1
205	0.104	0.167	0.042	0.065	0.143	0.104	0.146	0.109	0.13	0.021	0.208	0.13	0.087	0.136	0.167	0.146	0.146	0.125	-	0.159	0.104	0.104	0.083	0.087	0.083	0.071	0.174	0.065	0.104	0.167	0.087	0.125	0.083	0.25	0.021	0.104	0.125	0.063	0.136	0.125	0.068	0.125	0.083	0.146	0.109	0.091	-	-	-	-	-	-	-	-	-	-	0.042	0.045	-	-	0.021	-	-	0.021	-	-	0.022	-	-	-	0.021	-	-	-	0.023	-	0.023	0.022	0.021	-	-	0.021	-	-	-	-	-	-
207	0.417	0.458	0.458	0.5	0.381	0.438	0.396	0.391	0.348	0.417	0.333	0.261	0.457	0.432	0.292	0.438	0.354	0.35	0.364	0.432	0.479	0.417	0.417	0.522	0.521	0.571	0.304	0.435	0.313	0.354	0.348	0.375	0.375	0.333	0.521	0.396	0.396	0.333	0.432	0.438	0.5	0.375	0.5	0.479	0.348	0.455	-	-	-	-	-	0.114	0.104	-	-	-	0.042	0.068	0.043	-	-	-	-	0.021	-	0.023	0.065	-	-	-	0.042	0.021	-	0.043	0.023	-	0.023	0.022	0.042	-	0.021	0.021	0.045	-	-	-	0.023	-
209	-	-	-	-	0.024	-	-	0.022	-	-	-	-	-	-	-	-	-	-	-	0.023	-	-	-	-	-	-	-	-	-	-	-	-	-	-	0.021	-	-	-	-	-	0.023	-	-	-	-	-	-	-	-	-	-	-	-	-	-	-	-	-	-	-	-	-	-	-	-	-	-	-	-	-	-	-	-	-	-	-	-	-	-	-	-	-	-	-	-	-	-	-
Locus 27																																																																																											
244	0.438	0.292	0.417	0.326	0.262	0.313	0.458	0.479	0.5	0.271	0.313	0.304	0.457	0.477	0.396	0.5	0.5	0.357	0.457	0.477	0.354	0.478	0.25	0.304	0.479	0.381	0.348	0.391	0.417	0.354	0.435	0.375	0.375	0.271	0.396	0.438	0.417	0.333	0.5	0.354	0.205	0.396	0.333	0.375	0.37	0.318	0.729	0.833	0.813	0.761	0.667	0.591	0.75	0.833	0.826	0.813	0.667	0.652	0.739	0.522	0.583	0.955	0.708	0.696	0.625	0.727	0.609	0.833	0.646	0.708	0.667	0.729	0.667	0.674	0.705	0.708	0.705	0.413	0.396	0.783	0.833	0.583	0.636	0.773	0.69	0.771	0.636	0.75
248	0.563	0.708	0.583	0.674	0.738	0.688	0.542	0.521	0.5	0.729	0.688	0.696	0.543	0.523	0.604	0.5	0.5	0.643	0.543	0.523	0.646	0.522	0.729	0.696	0.521	0.619	0.652	0.609	0.583	0.646	0.565	0.625	0.625	0.729	0.604	0.542	0.583	0.667	0.5	0.646	0.795	0.604	0.667	0.625	0.63	0.682	0.271	0.167	0.188	0.239	0.333	0.409	0.25	0.167	0.174	0.188	0.333	0.348	0.261	0.478	0.417	0.045	0.292	0.304	0.375	0.273	0.391	0.167	0.354	0.292	0.333	0.271	0.333	0.326	0.295	0.292	0.295	0.587	0.604	0.217	0.167	0.417	0.364	0.227	0.31	0.229	0.364	0.25
250	-	-	-	-	-	-	-	-	-	-	-	-	-	-	-	-	-	-	-	-	-	-	0.021	-	-	-	-	-	-	-	-	-	-	-	-	0.021	-	-	-	-	-	-	-	-	-	-	-	-	-	-	-	-	-	-	-	-	-	-	-	-	-	-	-	-	-	-	-	-	-	-	-	-	-	-	-	-	-	-	-	-	-	-	-	-	-	-	-	-
Locus 32																																																																																										
98	0.375	0.375	0.396	0.435	0.643	0.417	0.333	0.313	0.386	0.229	0.417	0.435	0.391	0.409	0.458	0.5	0.438	0.333	0.587	0.409	0.5	0.5	0.304	0.413	0.521	0.452	0.457	0.478	0.521	0.354	0.609	0.479	0.333	0.479	0.354	0.521	0.458	0.208	0.417	0.479	0.348	0.458	0.563	0.313	0.435	0.318	-	0.229	0.208	-	0.125	0.048	0.021	0.125	0.022	0.208	0.146	0.13	0.043	0.087	0.063	-	0.104	0.125	0.188	0.068	0.065	-	-	-	0.063	0.042	0.021	0.152	0.068	-	0.023	0.022	0.208	-	0.021	0.208	0.273	0.023	0.048	-	0.023	0.063
100	0.625	0.604	0.604	0.565	0.333	0.583	0.667	0.667	0.614	0.771	0.583	0.522	0.565	0.591	0.542	0.5	0.542	0.619	0.413	0.591	0.5	0.5	0.696	0.587	0.479	0.548	0.543	0.522	0.479	0.646	0.391	0.521	0.646	0.521	0.625	0.479	0.542	0.792	0.563	0.521	0.63	0.542	0.438	0.667	0.522	0.682	1	0.771	0.792	1	0.875	0.952	0.979	0.875	0.978	0.792	0.854	0.87	0.957	0.913	0.938	1	0.896	0.875	0.813	0.932	0.935	1	1	1	0.938	0.958	0.979	0.848	0.932	1	0.977	0.978	0.792	1	0.958	0.792	0.727	0.932	0.952	1	0.977	0.938
102	-	0.021	-	-	0.024	-	-	0.021	-	-	-	0.043	0.043	-	-	-	0.021	0.048	-	-	-	-	-	-	-	-	-	-	-	-	-	-	0.021	-	0.021	-	-	-	0.021	-	0.022	-	-	0.021	0.043	-	-	-	-	-	-	-	-	-	-	-	-	-	-	-	-	-	-	-	-	-	-	-	-	-	-	-	-	-	-	-	-	-	-	-	0.021	-	-	0.045	-	-	-	-
Locus 34																																																																																										
104	0.083	0.104	0.021	0.087	0.119	0.063	0.188	0.146	0.065	0.167	0.146	0.022	0.087	0.159	0.063	0.063	0.104	0.143	0.109	0.136	0.104	0.125	0.104	0.022	0.083	0.048	0.043	0.043	0.146	0.083	0.109	-	0.104	0.125	0.125	0.083	0.167	0.125	0.125	0.125	0.196	0.104	0.063	0.146	0.022	0.045	-	-	-	-	-	-	0.021	-	-	-	-	-	0.022	0.022	0.125	-	0.042	0.063	-	0.114	0.022	-	-	-	0.021	0.042	-	-	0.068	-	-	-	0.063	0.043	-	0.042	-	-	0.024	-	0.068	-
110	0.917	0.896	0.938	0.87	0.81	0.875	0.771	0.854	0.913	0.771	0.854	0.978	0.87	0.818	0.875	0.833	0.833	0.833	0.891	0.841	0.896	0.813	0.875	0.957	0.875	0.905	0.957	0.891	0.854	0.875	0.891	0.958	0.854	0.875	0.875	0.833	0.813	0.854	0.875	0.833	0.761	0.896	0.896	0.833	0.957	0.932	1	1	1	1	1	1	0.958	1	1	1	1	1	0.978	0.978	0.875	1	0.958	0.938	1	0.886	0.978	1	1	0.979	0.979	0.958	1	1	0.932	1	1	1	0.938	0.957	0.958	0.958	1	1	0.976	0.979	0.932	1
112	-	-	0.042	0.043	0.071	0.063	0.042	-	0.022	0.063	-	-	0.043	0.023	0.063	0.104	0.063	0.024	-	0.023	-	0.063	0.021	0.022	0.042	0.048	-	0.065	-	0.042	-	0.042	0.042	-	-	0.083	0.021	0.021	-	0.042	0.043	-	0.042	0.021	0.022	0.023	-	-	-	-	-	-	0.021	-	-	-	-	-	-	-	-	-	-	-	-	-	-	-	-	0.021	-	-	-	-	-	-	-	-	-	-	0.042	-	-	-	-	0.021	-	-
Locus 44																																																																																										
180	-	-	-	-	-	-	-	-	-	-	-	-	-	-	-	-	0.042	-	-	-	-	-	-	-	-	-	-	-	-	-	-	-	-	-	-	-	-	-	-	-	-	-	-	-	-	-	-	-	-	-	-	-	-	-	-	-	-	-	-	-	-	-	-	-	-	-	-	-	-	-	-	-	-	-	-	-	-	-	-	-	-	-	-	-	-	-	-	-
182	0.792	0.813	0.833	0.63	0.833	0.729	0.667	0.813	0.87	0.708	0.667	0.848	0.826	0.727	0.563	0.667	0.583	0.833	0.739	0.795	0.625	0.75	0.813	0.783	0.75	0.905	0.761	0.739	0.646	0.833	0.63	0.75	0.875	0.833	0.625	0.75	0.813	0.75	0.826	0.771	0.717	0.792	0.583	0.792	0.761	0.886	0.563	1	1	0.957	0.938	0.957	0.875	0.958	0.957	0.958	0.854	0.804	0.957	0.978	0.938	0.977	0.938	0.854	1	0.773	0.935	1	1	1	0.917	0.896	1	0.913	1	1	1	0.957	0.896	0.913	1	0.896	0.932	0.977	0.929	0.958	0.932	1
184	0.208	0.188	0.167	0.37	0.167	0.271	0.333	0.167	0.043	0.271	0.333	0.13	0.174	0.273	0.396	0.333	0.313	0.167	0.261	0.205	0.333	0.25	0.188	0.196	0.25	0.095	0.239	0.239	0.292	0.167	0.283	0.229	0.125	0.167	0.375	0.25	0.188	0.229	0.109	0.208	0.239	0.208	0.417	0.208	0.239	0.114	-	-	-	0.043	0.021	0.043	0.083	-	-	0.021	-	0.065	-	-	0.042	-	0.021	0.104	-	0.045	-	-	-	-	0.021	0.021	-	0.022	-	-	-	0.022	0.021	-	-	-	-	0.023	-	-	0.023	-
186	-	-	-	-	-	-	-	-	-	-	-	-	-	-	-	-	0.042	-	-	-	-	-	-	-	-	-	-	-	-	-	-	0.021	-	-	-	-	-	-	0.022	-	0.043	-	-	-	-	-	-	-	-	-	-	-	-	-	-	-	-	-	-	-	-	-	-	-	-	-	-	-	-	-	-	-	-	-	-	-	-	-	-	-	-	-	-	-	-	-	-	-
188	-	-	-	-	-	-	-	0.021	0.087	0.021	-	0.022	-	-	-	0.042	0.021	-	-	-	0.021	-	-	0.022	-	-	-	0.022	0.063	-	0.087	-	-	-	-	-	-	0.021	0.043	0.021	-	-	-	-	-	-	0.438	-	-	-	0.042	-	0.042	0.042	0.043	0.021	0.146	0.13	0.043	0.022	0.021	0.023	0.042	0.042	-	0.182	0.065	-	-	-	0.063	0.083	-	0.065	-	-	-	0.022	0.083	0.087	-	0.104	0.068	-	0.071	0.042	0.045	-
190	-	-	-	-	-	-	-	-	-	-	-	-	-	-	-	-	-	-	-	-	0.021	-	-	-	-	-	-	-	-	-	-	-	-	-	-	-	-	-	-	-	-	-	-	-	-	-	-	-	-	-	-	-	-	-	-	-	-	-	-	-	-	-	-	-	-	-	-	-	-	-	-	-	-	-	-	-	-	-	-	-	-	-	-	-	-	-	-	-
Locus 46																																																																																										
223	0.375	0.167	0.25	0.273	0.238	0.271	0.188	0.333	0.227	0.271	0.167	0.239	0.217	0.341	0.25	0.188	0.125	0.333	0.457	0.273	0.188	0.188	0.188	0.37	0.292	0.167	0.348	0.174	0.146	0.333	0.196	0.146	0.188	0.125	0.25	0.208	0.229	0.292	0.25	0.146	0.152	0.146	0.375	0.25	0.109	0.159	0.688	0.833	0.833	0.804	0.833	0.841	0.521	0.771	0.804	0.833	0.625	0.565	0.674	0.522	0.542	0.614	0.646	0.625	0.583	0.614	0.5	0.792	0.771	0.708	0.625	0.833	0.583	0.739	0.545	0.854	0.75	0.457	0.333	0.696	0.688	0.792	0.773	0.818	0.357	0.896	0.545	0.792
224	0.625	0.833	0.75	0.727	0.762	0.729	0.813	0.667	0.773	0.729	0.833	0.761	0.783	0.659	0.75	0.813	0.875	0.667	0.543	0.727	0.813	0.792	0.813	0.63	0.708	0.833	0.652	0.826	0.854	0.667	0.804	0.854	0.813	0.875	0.75	0.792	0.771	0.708	0.75	0.854	0.848	0.854	0.625	0.75	0.891	0.841	0.313	0.167	0.167	0.196	0.167	0.159	0.479	0.229	0.196	0.167	0.375	0.435	0.326	0.478	0.458	0.386	0.354	0.375	0.417	0.386	0.5	0.208	0.229	0.292	0.375	0.167	0.417	0.261	0.455	0.146	0.25	0.543	0.667	0.304	0.313	0.208	0.227	0.182	0.643	0.104	0.455	0.208
227	-	-	-	-	-	-	-	-	-	-	-	-	-	-	-	-	-	-	-	-	-	0.021	-	-	-	-	-	-	-	-	-	-	-	-	-	-	-	-	-	-	-	-	-	-	-	-	-	-	-	-	-	-	-	-	-	-	-	-	-	-	-	-	-	-	-	-	-	-	-	-	-	-	-	-	-	-	-	-	-	-	-	-	-	-	-	-	-	-
Locus 50																																																																																										
254	-	-	-	-	-	-	-	-	-	-	-	-	-	-	-	-	-	0.024	-	-	-	-	-	-	-	-	-	-	-	-	-	-	-	-	-	-	-	-	0.042	-	0.022	-	-	-	-	-	0.021	-	-	-	-	0.045	0.021	-	-	-	-	-	-	-	-	-	-	-	-	-	-	-	-	-	-	-	-	-	-	-	-	-	-	-	-	-	-	-	-	-	-	-
258	0.771	0.854	0.646	0.841	0.905	0.875	0.875	0.854	0.795	0.771	0.875	0.913	0.87	0.864	0.75	0.854	0.813	0.857	0.739	0.886	0.896	0.813	0.896	0.848	0.896	0.833	0.804	0.87	0.75	0.896	0.739	0.875	0.875	0.854	0.854	0.75	0.833	0.813	0.792	0.875	0.804	0.813	0.917	0.826	0.891	0.75	0.979	1	1	1	1	0.955	0.979	1	1	0.979	0.979	1	1	1	1	1	1	1	1	1	1	1	1	1	1	1	1	0.978	1	1	1	1	1	1	0.979	1	1	1	1	1	1	1
262	0.021	-	-	-	-	-	-	0.021	0.023	-	0.042	-	-	-	-	-	-	-	-	-	-	0.021	-	0.022	-	-	-	-	-	0.021	0.022	-	-	-	-	0.021	-	-	0.021	0.021	-	-	0.021	-	-	-	-	-	-	-	-	-	-	-	-	-	-	-	-	-	-	-	-	-	-	-	-	-	-	-	-	-	-	-	-	-	-	-	-	-	-	-	-	-	-	-	-	-
266	0.063	0.104	0.146	0.068	-	0.104	0.042	0.063	0.091	0.104	0.042	0.043	0.065	0.045	0.083	0.042	0.104	0.024	0.13	0.068	0.042	0.063	0.063	0.065	0.063	0.024	0.13	0.065	0.021	0.042	0.13	0.063	0.042	0.021	0.063	0.063	0.083	0.063	0.104	0.063	0.065	0.083	0.021	0.065	0.043	0.136	-	-	-	-	-	-	-	-	-	0.021	0.021	-	-	-	-	-	-	-	-	-	-	-	-	-	-	-	-	-	-	-	-	-	-	-	-	-	-	-	-	-	-	-
270	0.021	-	0.042	0.023	0.024	-	-	-	-	0.021	-	-	-	-	0.021	-	-	-	-	0.023	-	-	-	-	-	-	-	-	-	-	-	-	-	-	-	-	-	-	-	-	0.022	-	-	0.043	-	-	-	-	-	-	-	-	-	-	-	-	-	-	-	-	-	-	-	-	-	-	-	-	-	-	-	-	-	-	-	-	-	-	-	-	-	-	-	-	-	-	-	-
274	-	-	-	-	0.024	-	-	-	-	-	-	-	-	-	-	-	-	-	-	-	-	-	-	-	-	-	-	-	-	-	-	-	-	-	-	-	-	-	-	0.021	-	-	-	-	-	-	-	-	-	-	-	-	-	-	-	-	-	-	-	-	-	-	-	-	-	-	-	-	-	-	-	-	-	-	-	-	-	-	-	-	-	-	-	-	-	-	-	-
278	-	-	-	-	-	-	-	-	-	-	-	-	-	-	-	-	-	-	-	-	-	-	-	-	-	-	-	-	0.021	-	-	-	-	-	-	-	-	-	-	-	-	-	-	-	-	-	-	-	-	-	-	-	-	-	-	-	-	-	-	-	-	-	-	-	-	-	-	-	-	-	-	-	-	-	-	-	-	-	-	-	-	-	-	-	-	-	-	-
280	-	-	-	-	-	-	-	-	-	-	-	-	-	-	-	-	-	-	-	-	-	-	-	0.022	-	-	-	-	0.021	0.021	-	-	-	-	-	-	-	-	-	-	-	0.021	-	-	-	-	-	-	-	-	-	-	-	-	-	-	-	-	-	-	-	-	-	-	-	-	-	-	-	-	-	-	-	-	-	-	-	-	-	-	0.021	-	-	-	-	-	-	-
282	-	-	-	-	-	-	-	-	-	-	-	-	0.022	-	-	-	-	-	-	-	-	-	-	-	0.021	0.024	-	-	-	-	-	0.021	-	-	0.021	-	-	-	-	-	-	-	-	-	0.022	0.023	-	-	-	-	-	-	-	-	-	-	-	-	-	-	-	-	-	-	-	-	-	-	-	-	-	-	-	-	-	-	-	-	-	-	-	-	-	-	-	-	-	-
286	0.104	0.042	0.146	0.068	0.048	0.021	0.083	0.063	0.068	0.104	0.042	0.022	0.043	0.091	0.125	0.104	0.083	0.095	0.13	0.023	0.042	0.083	0.021	0.022	0.021	0.119	0.065	0.065	0.167	0.021	0.109	0.042	0.083	0.125	0.063	0.146	0.083	0.125	0.042	0.021	0.087	0.083	0.042	0.065	0.043	0.091	-	-	-	-	-	-	-	-	-	-	-	-	-	-	-	-	-	-	-	-	-	-	-	-	-	-	-	0.022	-	-	-	-	-	-	-	-	-	-	-	-	-	-
290	0.021	-	0.021	-	-	-	-	-	0.023	-	-	0.022	-	-	0.021	-	-	-	-	-	0.021	-	-	0.022	-	-	-	-	0.021	-	-	-	-	-	-	0.021	-	-	-	-	-	-	-	-	-	-	-	-	-	-	-	-	-	-	-	-	-	-	-	-	-	-	-	-	-	-	-	-	-	-	-	-	-	-	-	-	-	-	-	-	-	-	-	-	-	-	-	-
292	-	-	-	-	-	-	-	-	-	-	-	-	-	-	-	-	-	-	-	-	-	0.021	-	-	-	-	-	-	-	-	-	-	-	-	-	-	-	-	-	-	-	-	-	-	-	-	-	-	-	-	-	-	-	-	-	-	-	-	-	-	-	-	-	-	-	-	-	-	-	-	-	-	-	-	-	-	-	-	-	-	-	-	-	-	-	-	-	-
294	-	-	-	-	-	-	-	-	-	-	-	-	-	-	-	-	-	-	-	-	-	-	0.021	-	-	-	-	-	-	-	-	-	-	-	-	-	-	-	-	-	-	-	-	-	-	-	-	-	-	-	-	-	-	-	-	-	-	-	-	-	-	-	-	-	-	-	-	-	-	-	-	-	-	-	-	-	-	-	-	-	-	-	-	-	-	-	-	-
Summary																																																																																										
H_o	0.301	0.265	0.301	0.325	0.303	0.315	0.289	0.285	0.299	0.321	0.328	0.255	0.301	0.308	0.301	0.298	0.295	0.271	0.318	0.308	0.265	0.305	0.34	0.304	0.289	0.279	0.296	0.292	0.321	0.277	0.311	0.295	0.354	0.298	0.295	0.348	0.256	0.277	0.268	0.322	0.286	0.292	0.301	0.272	0.28	0.266	0.155	0.083	0.104	0.081	0.089	0.101	0.119	0.11	0.096	0.125	0.205	0.234	0.125	0.152	0.152	0.071	0.137	0.18	0.119	0.172	0.18	0.06	0.065	0.098	0.155	0.158	0.119	0.168	0.182	0.071	0.117	0.127	0.217	0.13	0.116	0.196	0.182	0.107	0.122	0.074	0.146	0.077
H_o (SD)	0.212	0.178	0.194	0.142	0.192	0.21	0.18	0.19	0.189	0.162	0.22	0.162	0.182	0.216	0.193	0.195	0.183	0.189	0.186	0.153	0.182	0.175	0.209	0.212	0.195	0.164	0.166	0.154	0.172	0.199	0.176	0.179	0.202	0.205	0.197	0.22	0.181	0.13	0.177	0.202	0.153	0.166	0.193	0.164	0.179	0.202	0.272	0.139	0.18	0.155	0.151	0.134	0.111	0.161	0.145	0.142	0.23	0.203	0.175	0.194	0.148	0.129	0.176	0.168	0.199	0.156	0.222	0.112	0.107	0.162	0.168	0.17	0.208	0.191	0.171	0.148	0.164	0.165	0.181	0.159	0.172	0.158	0.208	0.196	0.133	0.131	0.153	0.149
H_e	0.344	0.303	0.345	0.359	0.329	0.35	0.345	0.309	0.332	0.34	0.335	0.292	0.343	0.37	0.349	0.35	0.356	0.335	0.351	0.346	0.315	0.339	0.335	0.325	0.341	0.314	0.356	0.331	0.352	0.311	0.353	0.323	0.331	0.32	0.334	0.344	0.315	0.33	0.335	0.347	0.346	0.338	0.338	0.32	0.325	0.293	0.144	0.094	0.108	0.101	0.12	0.164	0.174	0.117	0.11	0.128	0.2	0.227	0.152	0.161	0.17	0.084	0.155	0.188	0.134	0.193	0.176	0.075	0.115	0.114	0.17	0.154	0.127	0.165	0.186	0.088	0.131	0.149	0.214	0.134	0.117	0.203	0.168	0.105	0.152	0.078	0.168	0.093
H_e (SD)	0.171	0.186	0.188	0.149	0.159	0.174	0.184	0.185	0.177	0.157	0.173	0.18	0.165	0.17	0.177	0.164	0.181	0.18	0.164	0.169	0.176	0.17	0.134	0.188	0.165	0.17	0.169	0.164	0.173	0.18	0.155	0.169	0.157	0.181	0.158	0.185	0.184	0.133	0.196	0.177	0.158	0.163	0.194	0.178	0.19	0.183	0.211	0.143	0.166	0.168	0.179	0.166	0.167	0.161	0.156	0.133	0.198	0.19	0.168	0.208	0.184	0.161	0.179	0.185	0.211	0.177	0.183	0.13	0.182	0.189	0.19	0.161	0.203	0.162	0.177	0.174	0.18	0.195	0.165	0.168	0.169	0.159	0.188	0.164	0.186	0.126	0.185	0.16
P_{total}	43.21	40.74	41.98	38.27	41.98	40.74	40.74	40.74	44.44	43.21	40.74	40.74	44.44	40.74	39.51	35.8	44.44	45.68	37.04	41.98	43.21	43.21	44.44	40.74	40.74	41.98	37.04	39.51	40.74	39.51	40.74	40.74	44.44	35.8	41.98	38.27	38.27	40.74	39.51	44.44	43.21	38.27	40.74	41.98	39.51	37.04	17.28	14.81	12.35	17.28	17.28	33.33	35.8	17.28	19.75	25.93	27.16	30.86	27.16	24.69	25.93	14.81	24.69	28.4	14.81	30.86	30.86	12.35	13.58	16.05	28.4	30.86	16.05	29.63	32.1	9.88	23.46	27.16	34.57	22.22	23.46	33.33	24.69	16.05	24.69	17.28	28.4	12.35
P_{95}	43.21	38.27	40.74	38.27	41.98	39.51	37.04	38.27	44.44	43.21	39.51	39.51	41.98	38.27	38.27	35.8	44.44	43.21	35.8	40.74	40.74	43.21	44.44	38.27	38.27	39.51	35.8	39.51	39.51	38.27	39.51	38.27	43.21	34.57	41.98	38.27	34.57	39.51	39.51	41.98	43.21	38.27	39.51	40.74	37.04	37.04	13.58	12.35	12.35	12.35	16.05	25.93	28.4	14.81	16.05	20.99	24.69	28.4	19.75	18.52	23.46	9.88	22.22	25.93	12.35	27.16	25.93	9.88	12.35	13.58	24.69	22.22	13.58	23.46	28.4	8.64	17.28	20.99	33.33	17.28	17.28	28.4	19.75	14.81	18.52	12.35	24.69	11.11

20-24 individuals were sequenced for all loci and all populations.
Table produced with the help of Pop100Gene [Piry S and Bouget C (1999) Available from http://www1.montpellier.inra.fr/URLB/pop100gene/pop100gene.html]

Alb - plots in the Schwäbische Alb
Sch - plots in the Schorfheide-Chorin

Unique allele
Private allele
