# Supplementary material for: Living in Heterogeneous Woodlands – Are Habitat Continuity or Quality Drivers of Genetic Variability in a Flightless Ground Beetle?
Source: PLoS One. 2015 Dec 7;10(12):e0144217. doi: 10.1371/journal.pone.0144217 (PMC4671619; doi:10.1371/journal.pone.0144217)
Supplement: S1 Text — (DOCX) [file pone.0144217.s012.docx]

## Supplementary methods

Number of *Abax parallelepipedus* individuals (2008): For estimating local population densities on each plot we used pitfall traps (funnel traps with a diameter of 15 cm; see [1] for general design). We installed three pitfall traps per experimental plot, one in each of three randomly selected corners. As sampling fluid we used copper-sulfate solution (3%) and added a drop of detergent to reduce surface tension. During the entire growing season from mid of April to mid of October 2008 we emptied traps five times in an interval of five to six weeks. We transferred arthropods into 70% ethanol in the field and subsequently sorted two out of three randomly selected trap samples per months to taxonomic orders in the laboratory (for details see [2]). All carabids were identified to species level by a taxonomic specialist.

Vegetation sampling (percent of closed forest species, litter cover, cover of trees (>10m), cover of deadwood, number of vascular plant species): In 2009, we recorded the vegetation of all plots in the summer, in an area of 20 m × 20 m. We identified all vascular plants and estimated the percentage cover per species separately for two tree layers (5–10 m and >10 m), the shrub layer (including all woody species older than one year 0–5 m), and the herb layer (including phanerophyte seedlings). In addition, we identified all bryophyte and lichen species in all plots. Furthermore, we estimated the percentage cover of deadwood and litter. See [3] and [4].

Percentage of closed forest species: We classified all of the species found in the vegetation surveys according to Schmidt et al. [5]. We then calculated what percentage found of all species are defined as closed species (category 1.1).

Percentage of surrounding landscape which is forested: Forested areas were defined as those covered by forests (OTF_Wald_Forst) or thick groves (OTF_Gehölz) according to the ATKIS Basis - Digitales Landschaftsmodell maps from the year 2010 (Arbeitsgemeinschaft der Vermessungsverwaltungen der Länder der Bundesrepublik Deutschland). Using ArcGIS ver. 9.3, a buffer of two kilometers was defined surrounding each plot and the percentage of the total area covered which is forested was calculated.

Soil data: At each of the 88 plots we took 14 samples of the organic layers (O_i_, O_e_ and O_a_ horizon) with a 400 cm^2^ metal frame along two 40 m transects. The thickness of the different organic layers was measured with a ruler at each of the 14 samplings points and an average thickness per plot was calculated. Afterwards, a composite sample for the O_i_, O_e_ and O_a_ horizon of each plot was prepared by mixing the samples from the 14 sampling points. At each plot, we also collected 14 mineral soil cores with a split tube sampler (diameter of 5 cm) at the same sampling points where the organic layer was removed. We then prepared a composite sample for the mineral soil by mixing the upper 10 cm of the 14 mineral soil cores. For further analyses, all composite organic layer samples were dried at a temperature of 60°C and all composite mineral soil samples were dried at a temperature of 40°C. All mineral soil samples were additionally sieved to <2 mm. We determined the pH values of the sieved mineral soil samples in duplicate using 0.01 M CaCl2 solution and a soil solution ratio of 1:2.5. Prior to C and N analysis, subsamples of the organic horizons and of the mineral soil were ground in a ball mill (RETSCH MM200, Retsch, Haan, Germany). Total C and N concentrations of organic layers and the mineral soil were determined by dry combustion with an elemental analyzer (VarioMax, Hanau, Germany). Inorganic C in mineral soils was measured after removing all organic carbon at a temperature of 450 °C for 16 hours. Organic carbon concentrations in the mineral soil result from the difference between total and inorganic carbon concentrations. See also [6].

1. Lange M, Gossner MM, Weisser WW (2011) Effect of pitfall trap type and diameter on vertebrate by-catches and ground beetle (Coleoptera: Carabidae) and spider (Araneae) sampling. Methods Ecol Evol 2: 185-190.

2. Lange M, Türke M, Pašalić E, Boch S, Hessenmöller D, Müller J, Prati D, Socher SA, Fischer M, Weisser WW, Gossner MM (2014) Effects of forest management on ground-dwelling beetles (Coleoptera; Carabidae, Staphylinidae) in Central Europe are mainly mediated by changes in forest structure. For Ecol Manage 329: 166-176.

3. Boch S, Prati D, Müller J, Socher SA, Baumbach H, Buscot F, Gockel S, Hemp A, Hessenmöller D, Kalko EKV, Linsenmair KE, Pfeiffer S, Pommer U, Schöning I, Schulze E-D, Seilwinder C, Weisser WW, Wells K, Fischer M (2013) High plant species richness indicates management-related disturbances, rather than the conservation status of forests. Basic Appl Ecol 14: 496–505.

4. Boch S, Prati D, Hessenmöller D, Schulze ED, Fischer M (2013) Richness of lichen species, especially of threatened ones, is promoted by management methods furthering stand continuity. PLoS ONE 8(1): e55461 doi: 10.1371/journal.pone.0055461.

5. Schmidt M, Kriebitzsch WU, Ewald J (2011) Waldartenlisten der Farn-und Blütenpflanzen, Moose und Flechten Deutschlands. Bonn: Bundesamt für Naturschutz. 111 p.

6. Solly EF, Schöning I, Boch S, Kandeler E, Marhan S, et al. (2014) Factors controlling decomposition rates of fine root litter in temperate forests and grasslands. Plant Soil 382: 203-218.
